# Integrating protein copy numbers with interaction networks to quantify stoichiometry in clathrin-mediated endocytosis

**DOI:** 10.1038/s41598-022-09259-w

**Published:** 2022-03-30

**Authors:** Daisy Duan, Meretta Hanson, David O. Holland, Margaret E. Johnson

**Affiliations:** 1 TC Jenkins Department of Biophysics, Johns Hopkins University, 3400 N Charles St, Baltimore, MD 21218 USA; 2 NIH, Bethesda, MD 20892 USA

**Keywords:** Computational biophysics, Endocytosis

## Abstract

Proteins that drive processes like clathrin-mediated endocytosis (CME) are expressed at copy numbers within a cell and across cell types varying from hundreds (e.g. auxilin) to millions (e.g. clathrin). These variations contain important information about function, but without integration with the interaction network, they cannot capture how supply and demand for each protein depends on binding to shared and distinct partners. Here we construct the interface-resolved network of 82 proteins involved in CME and establish a metric, a stoichiometric balance ratio (SBR), that quantifies whether each protein in the network has an abundance that is sub- or super-stoichiometric dependent on the global competition for binding. We find that highly abundant proteins (like clathrin) are super-stoichiometric, but that not all super-stoichiometric proteins are highly abundant, across three cell populations (HeLa, fibroblast, and neuronal synaptosomes). Most strikingly, within all cells there is significant competition to bind shared sites on clathrin and the central AP-2 adaptor by other adaptor proteins, resulting in most being in excess supply. Our network and systematic analysis, including response to perturbations of network components, show how competition for shared binding sites results in functionally similar proteins having widely varying stoichiometries, due to variations in both abundance and their unique network of binding partners.

## Introduction

The copy numbers of distinct proteins have been counted for a number of single cells^1–5^ and tissues^6,7^, where abundances can vary per cell type by at least six orders of magnitude. This protein copy number data is a valuable resource for identifying function and phenotype per cell. Highly abundant proteins, such as the chaperone ATPase HSPA8/HSC70, often exhibit broad functionality^8^. Protein copy numbers or expression profiles indicate variations from cell-type to cell-type, or between healthy and diseased cells^9^, where the genes that encode specific proteins are otherwise identical. However, pair correlations only scratch the surface of the information available. The relative abundance of proteins reflects a network of relationships. Can we use such a network description to capture the higher order connectivity and thus quantify the significance of copy number variations on function? In clathrin-mediated endocytosis (CME), an essential process for transport across the plasma membrane^10–14^, clathrin is a highly abundant trimeric protein complex, but it has dozens of binding partners that could leave it in short supply. Some proteins, such as dynamin 3, are only expressed in a subset of mammalian cell types, but given that dynamin proteins are encoded by three genes, the two other genes could compensate for this shortage. Here, we define a stoichiometric balance ratio (SBR) to provide a metric comparing stoichiometry of proteins in CME using a mathematical optimization that integrates both the protein network and the known copy numbers of proteins. The inclusion of the network allows us to identify correlated changes across cell types, where a reduced expression of one protein, for example, is compensated by reduced expression of a partner, or a partner’s partner. We identify proteins that are in excess supply (super-stoichiometric) relative to their partners, in all cell types, and vice-versa (sub-stoichiometric).

Previous networks of mammalian CME detailed which proteins interacted with one another, and with the key recruiting lipid PI(4,5)P_2_, but lacked the resolution of the interfaces or domains that mediate binding^15^. Because proteins typically contain multiple interfaces and can directly bind to one another in both competitive (shared interfaces) and cooperative (distinct interfaces) ways, the interface-resolved network is essential for assessing how protein copies can be partitioned between distinct interactions. Our network integrates previous work on the adaptor protein AP-2^15–17^ and clathrin^18^ network with our comprehensive literature curation here of over 280 publications on proteins chosen based on three studies of systems-level CME^10,11,19^, plus 5 lipids and 9 classes of transmembrane binding partners (referred to as cargo)^20,21^ (Tables S1–S3). Such a structurally-resolved network provides a rich resource on its own. The topology of structure-resolved networks is constrained to ensure specificity of protein–protein interactions, biasing select network motifs in the interface-interaction networks^19,22–24^ and the protein interaction networks^24^. The addition of protein structures expands on evolutionary growth mechanisms of protein–protein interactions ^25,26^ and provides a basis to start inferring temporal dynamics^27^. Structure-resolved networks provide templates for predicting specific and structurally resolved protein–protein interactions^28–30^. By identifying specific interfaces that mediate a majority of protein interactions, these maps can be useful for designing mutations that disrupt global function^30^. These networks quantitate domain participation, demonstrating that Short Linear Motifs (SLiMs)^31^ mediate more than half of the interactions across the CME network, which is markedly distinct from a signaling network^32^. A structure-resolved network is typically conserved across cell types, aside from a few changes in binding interactions due to isoform variations (e.g. Synaptojanin), and thus the primary changes in interactomes across cell type occur at protein abundance levels.

Total abundance of each protein in a single cell is a key factor in controlling function, because it controls binding and complex formation, with high abundance correlated with essentiality^33^. CME is a constantly ongoing process, where at any point in time in the cell, many clathrin-coated vesicles are being formed and trafficked within the cell^34^, resulting in proteins participating in a relatively uniform distribution throughout the full cell volume^34^. This means that at any lengthscale that exceeds that of a few vesicles, it is reasonable to assume the concentration of each protein is similar at one sub-volume relative to another. In other words, CME proteins typically do not exhibit a strong spatial gradient throughout the cell^5^. Total protein abundance thus controls the local concentration, with imbalances in copy numbers thus expected both globally and locally.

Comparing changes in each protein’s abundance from one cell type to another does not control for relationships between proteins that act as partners or homologs to one another. As we show below, only considering nearest neighbors to quantify stoichiometry, while a useful first measure of imbalances^32^, misses out on much of the redundancy and competition of interactions that influences demand for any protein. Earlier work was able to efficiently quantify equilibrium behavior within a network of protein interactions, but did not account for the multiple interfaces that each protein uses to mediate interactions^35^. While the most comprehensive method to integrate abundances with interaction networks is through mass-action kinetics (MAK) approaches to predict all bound and unbound states, networks of the scale constructed here have both too much and too little information to construct a tractable and predictive kinetic model focused on biological outcomes, rather than principles of network dynamics^36^. On one hand, the network lacks upstream and downstream interactions for the CME proteins that would be used in a whole-cell type model^37^. On the other hand, it has too many components and interactions (> 600) to create coarse-grained^38^ or sub-network^19,32,39^ kinetic models, particularly as vesicle formation depends not only on knowledge of all pairwise biochemical rates, but cooperativity, mechanics, and higher-order assembly. However, this structure-resolved network resolution is rich with detailed information on the extensive binding between CME proteins. Given that many abundances are also known by cell type, we thus perform an analysis that combines all of this information to quantify stoichiometric imbalances.

Therefore, to account for correlations due to interactions and redundancy between proteins, we use a model of stoichiometric balance that builds binding interactions into a quantitative comparison of relative protein copy numbers. For example, an obligate multi-protein complex (like the ribosome) in stoichiometric balance would have each protein subunit expressed at copy numbers relative to their stoichiometry in the complex. Our model thus operates on the hypothesis that balanced copies are desirable, and identifies where imbalances exist. Stoichiometric balance has the benefits of improving complex yield, by minimizing the formation of mis-interactions^22,23,40,41^, and by avoiding sequestering of subunits in incomplete complexes^42–44^. Experimental evidence supports stoichiometric balance for proteins involved in highly stable complex formation^2,45,46^. When extended to larger networks of reversible interactions, this idea must account for interfaces that have multiple competing partners^39^. Each *interface* of a protein must then be expressed to match (by summing over) all of its partner interfaces. Identifying whether a protein’s abundance is sub- or super-stoichiometric relative to its partners in a specific network is informative for function. Super-stoichiometric proteins can be beneficial to drive complex formation involving weak interactions, and can ultimately improve assembly yield^47^. Sub-stoichiometric proteins can be beneficial in selectively activating only one of many partners^32^. By comparing the relative stoichiometric abundance of our protein network across cell-types, we can evaluate whether any super- or sub-stoichiometry relative to partners is conserved.

An integrated and objective approach towards quantifying stoichiometry is useful in a system like CME, where dozens of components contribute to the formation of a protein coat for the purpose of capturing and internalizing cargo across the plasma membrane^11^. While any systems analysis within cell biology is necessarily incomplete, as we cannot assume every single component is known, there is a large collection of data on CME which we find sufficient to start quantifying stoichiometry. The sequence of events in CME have been broadly defined^10^ and we exclude downstream membrane trafficking. The central protein core of clathrin-coated vesicle formation is the trimeric clathrin complex, the adaptor protein complex AP-2, additional adaptor proteins (proteins that bind to cargo), and the GTPase dynamin. We expand to include lipids, internalized cargo, and key additional binding partners that primarily help remodel or enzymatically modify the membrane. The final network of 82 proteins^10,11,48^ plus cargo results in a unique binding network for most cargo adaptor proteins. We acknowledge that without considering the interactions of each protein in our network with all other cellular proteins, we cannot capture the full physiologic complexity of the cell, and the participation of proteins in pathways other than CME. We nonetheless use a protein’s total cellular abundance to define stoichiometry, in the absence of quantitative data on fractional availability of each protein for one pathway over another. Although the concentrations of many proteins contained within clathrin-coated vesicles has been determined^49^, this excludes many proteins that contribute to vesicle formation without being in the final vesicle, nor does it include comprehensive counts of cargo proteins.

In this paper, we have two parts. In the first part, we present and analyze the newly constructed interface-resolved interaction network for mammalian CME, and in the second part we analyze the abundances and stoichiometry of proteins across this network. Our structural and functional characterization of the network highlight how clathrin/AP-2 vs dynamin center two distinct modules in the CME network. Links between these modules are often supplied by interactions involving SH3 domains with proline-rich domains (PRD). In part two, we directly compare the total protein abundances collected from three distinct cell populations: a rat synaptosome (a small compartment isolated from the synaptic terminal of a neuron), a mouse fibroblast, and a human epithelial (HeLa) cell. Although the synaptosome is a compartment and not a true cell, we refer to it as a distinct cell ‘type’ as it originates from a neuron. This comparison highlights which proteins have highly disparate expressions. To introduce the coupling between interacting components, we first quantify stoichiometry for each protein relative to its direct partners, before expanding to the full network of proteins and cargo with the SBR metric defined below. By comparing the SBR across cell types and as each protein is removed from the network, we show how highly abundant proteins play a strong role in determining network stoichiometry, with highly connected proteins less significant overall. Importantly, with each perturbation to the network, including removal of all cytoskeletal components, we can quantify changes to the SBR of all proteins in the network, without needing to choose the response of a specific protein. Network perturbation also demonstrates how several stoichiometric trends are robust even with seemingly large changes to the interactome. We focus much of our quantitative comparisons on adaptor proteins and cargo, as they are functionally central and exhibit both common and diverse binding patterns that control their stoichiometry. Finally, we conclude with limitations and future directions made possible by the datasets generated and analyses performed here.

## Results

### The CME proteome and interactome is well defined structurally and functionally from mining extensive literature

**Figure 1:**
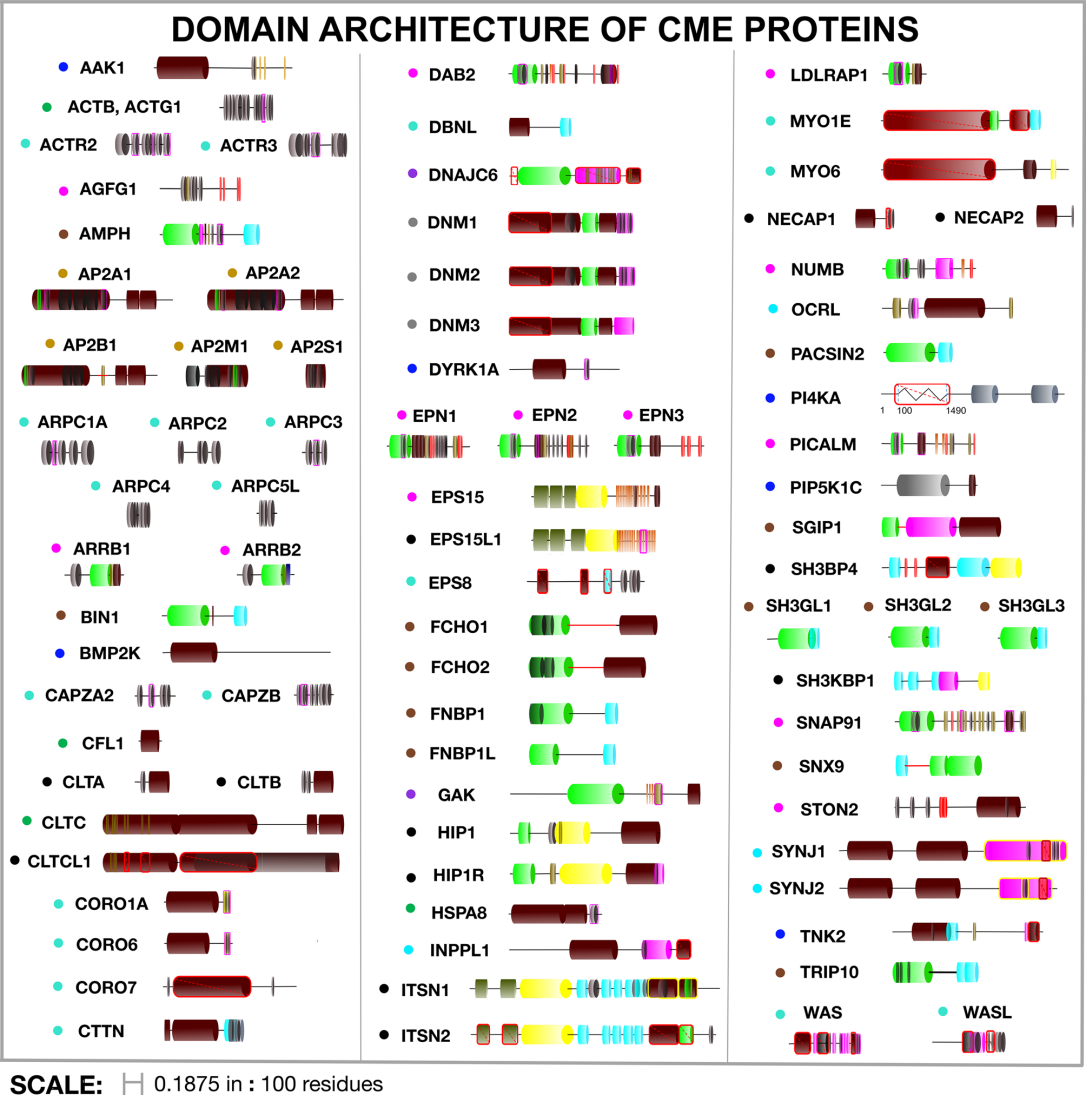
Domain and interface architecture of 82 CME proteins in our human interface-resolved protein network. Information on interfaces was curated from SMART^50^ and low-throughput studies, where structured domains are in some cases subdivided by residues that represent binding interfaces (e.g. the five AP-2 genes indicate where they bind the other A/α, B/β, S/σ, M/μ subunits). The colored dot next to each protein name indicates the class to which we have assigned it: the central AP-2 adaptor subunits in mustard yellow, other cargo adaptor proteins in pink, kinases in dark blue (which act on proteins and lipids), phosphatases in light blue (which act only on lipids), dynamins in gray (necessary for vesicle fission), enzyme co-factors of the ATPase chaperone protein HSC70/HSPA8 in purple (needed for disassembly of cages), BAR (bin/amphiphysin/rvs) domain proteins in brown, cytoskeletal proteins in mint, and a group of always-abundant proteins in green. Protein length is scaled to reflect residue length, with distinct domains shown in colors, including structured domains and SLiMs that mediate binding. Red outlines indicate domains that are not present in our network, as we did not find any interacting partners for them; it does not mean they cannot mediate any protein interactions. Domains are colored: SH3 (blue), PRD/proline-rich domain (pink), lipid binding (green), coiled-coil (yellow), other structured (brown).

**Figure 2:**
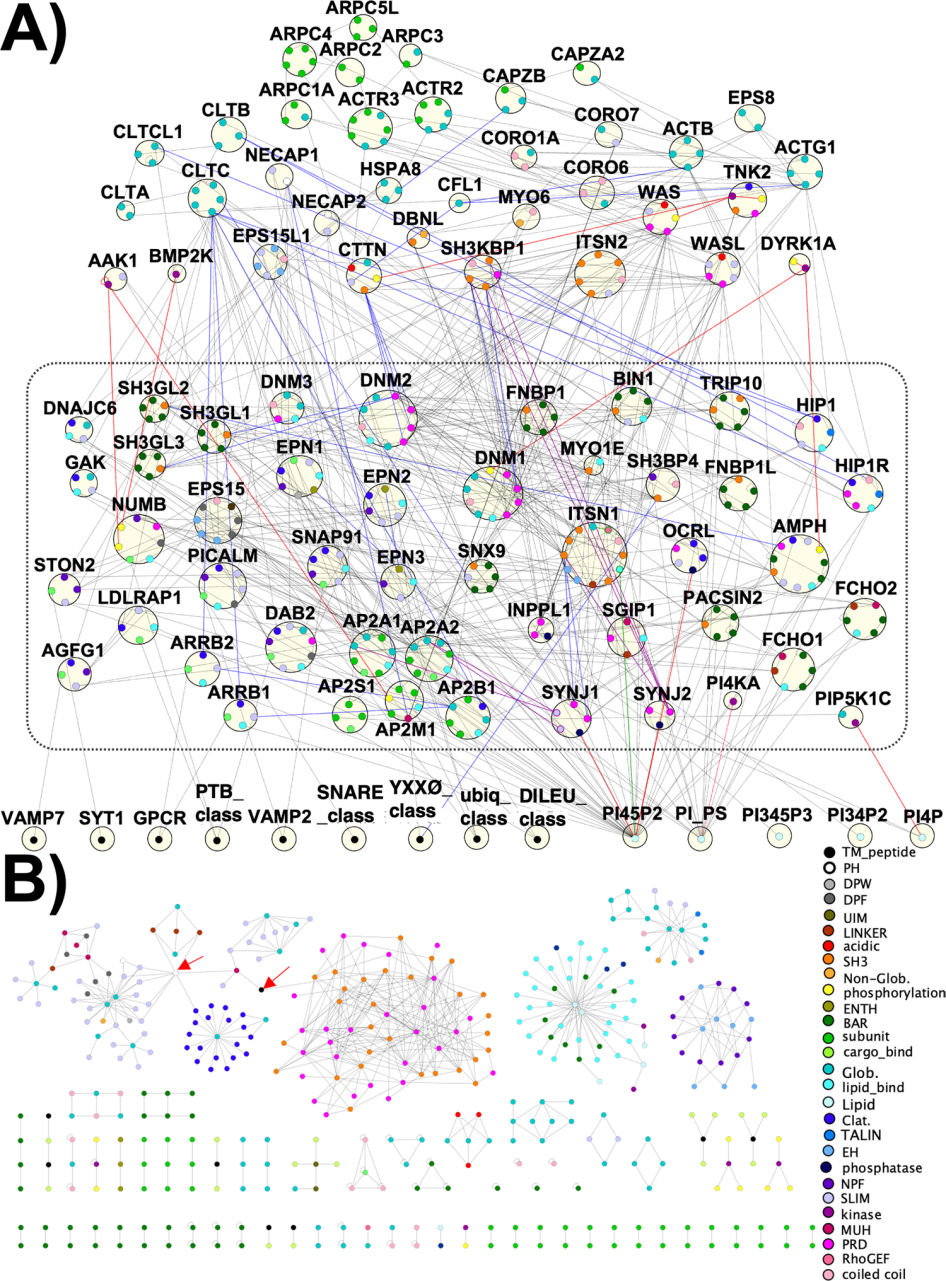
The interface-resolved PPIN for our mammalian CME proteome reveals extensive and redundant capacity to assemble and localize to the membrane. (**A**) The CME PPIN includes the 82 proteins from Fig. 1 labeled by gene name, as well as 5 phospholipids (PI(4,5)P_2_, a phosphatidylinositol/phosphaditylserine group/PI_PS, PI(3,4)P_2_, PI(4)P, and PI(3,4,5)P_3_) and 9 groups of transmembrane receptors or cargo along the bottom row, labeled by gene name or class/grouping. The box contains the set of proteins that directly bind to lipids and cargo types. Proteins that do not bind to lipids nor cargoes are located directly above the dashed black box. Unique interfaces are shown, color-labeled according to interface type. Red edges indicate enzymatic reactions, and blue edges indicate some conditionality or regulation of the interaction. Purple edges are isoform specific. (**B**) Map of interface-resolved PPIs in the network. When separated from the parent proteins, the interface-interaction network (IIN) illustrates the selectivity of binding pairs, visibly encoded in the highly disconnected modules. Within each module, interfaces typically interact in motifs that include selective pairs or pairs-of-pairs (square motifs), or with a central hub interface surrounded by multiple spokes of one specific domain. Red arrows indicate noted ‘bridging’ nodes. BAR: Bin-Amphiphysin-Rvs domain; CC: coiled coil; Clat.: clathrin-box motif; DPF: Aspartic Acid(D)-Proline(P)-Phenylalanine(F) motif; DPW: Aspartic Acid(D)-Proline(P)-Tryptophan(W) motif; EH: Eps15 Homology; NPF Asparagine(N) Proline(P) Phenylalanine(F) domain; F-BAR: FCH(F)-Bin-Amphiphysin-Rvs domain; ENTH/ANTH: Epsin N-terminal Homology/AP180 N-terminal Homology domain; Glob.: globular domain; PH: Pleckstrin-Homology domain; SH3: SRC Homology 3 domain PRD: Proline Rich Domain; SLiM: short linear motif; μHD: mu-homology domain.

We selected the 82 proteins in our CME cytoplasmic proteome using a previously curated yeast CME interactome ^51^, live-cell studies ^10,52^, and comprehensive reviews ^11^. We classified these proteins and colored them in the figures of this paper according to their primary known function in CME, except a set of proteins (clathrin heavy chain CLTC; actin genes ACTG1 and ACTB; ATPase HSPA8/HSC70; and cofilin CFL1) that are designated based on their conserved high abundance (Fig. 1). Our full network includes key plasma membrane phospholipids such as the essential PI(4,5)P_2_^53^, and transmembrane protein targets, visible as the bottom row of nodes in Fig. 2. We define nine groups of transmembrane receptor/cargo, based on their specificity of being bound by one or more adaptor proteins^20,21^. The SNARE_class, PTB_class, YXXØ_class, ubiq_class, and DILEU_class each contain multiple transmembrane proteins, whereas the other 4 are specific protein genes with distinct specificity (see Table S1 for all receptors). In our classification scheme, we separated the central cargo adaptor AP-2 (mustard yellow) from the other cargo adaptors (pink), because functionally, it is the most essential adaptor, without which cargo uptake is drastically reduced^54^, and topologically, it is a heterotetramer (encoded in 5 genes distinguished by subunit letters A/α, B/β, S/σ, M/μ, with two genes for the A/α adaptor) that acts as a hub in the network. Alternative to our functional classification, CME proteins can be classified based on their arrival time at sites of CME^10,11,55^.

We constructed the interface-resolved network (Fig. 2) with the proteins shown in Fig. 1 by downloading all documented protein–protein interactions from databases BioGrid^56^, IntAct^57^ and Mentha^58^, and assigning interfaces to each interaction if sufficient information was available. In “Methods”, we detail all steps taken to construct the network (see the flowchart in Fig. S1), including the addition of protein–lipid (previously compiled here in Ref.^59^) and protein–cargo interactions^20,21,60^, resulting in over 600 interface–resolved interactions with details from almost 300 publications. The proteins and copy numbers are compiled in Table S1, the binding domains in Table S2, and the full interface resolved network in Table S3.

### The interface–interaction network has a topology optimized for binding specificity

Our network has characteristics previously demonstrated to be beneficial for binding specificity, indicated directly by how the interface-interaction network (IIN) in Fig. 2B breaks into islands or modules by interface type. This includes a module mediated by Src Homology 3 (SH3) and Proline-Rich Domain (PRD) interactions (orange and pink module, Fig. 2B) that we discuss further below. This modularity is expected due to constraints on evolving interfaces both for specific interactions and against nonspecific interactions^22,23,51^, which further constrains the protein network^24^. The network topology thus displays an abundance of hub and square motifs^24^, in addition to pairs (bottom, Fig. 2B), where the motifs describe the shapes formed by the connected interfaces, and hub, square and pair motifs all support the complementary structure and chemistry needed for specificity^24^. The IIN contains few triangle motifs, which are difficult to optimize for specificity, unless the interfaces also bind to themselves^24^, which we see is true in Fig. 2B. We defined 28 classes of interface types, some of which are structured domains (e.g. SH3, BAR), but the majority of which represent short linear motifs (SLiMs) often found within disordered or unstructured regions of proteins (Fig. 2B). The modules do not all separate cleanly, and the bridging nodes often have unusual binding or regulatory behavior. For instance, SH3BP4 has an SH3 domain that uniquely binds the YXXØ_class cargo peptide (Fig. 2B, red arrow to black TM_peptide), connecting the SH3-PRD module to modules on its left. Similarly, the PH ear-domain of NECAP1 displays significant promiscuity in the types of partners it binds (Fig. 2B, red arrow to white PH domain), including SLiMs, linker regions, the clathrin-box motif of AP2B1, and the AP2M1 subunit. This promiscuity allows NECAP1 to localize to sites of CME, and then act as a negative regulator of AP-2 activity^61^.

### The CME network partitions via connectivity to the membrane

By arranging our network with the membrane cargo and lipids along the bottom, we can clearly see the extent to which the cytoplasmic proteins in CME directly localize to the membrane surface, as indicated by the boxed region in the center (Fig. 2A). These proteins also contain large numbers of PPIs, allowing them to form a large variety of connected networks with multiple links to the membrane surface. By localization to the membrane, these proteins can also exploit dimensional reduction in what is effectively the 2D space of the surface, which allows proteins to nucleate complexes at much lower concentrations than are required in solution^59^. This effect is powerful despite the fact that a primary lipid recruiter, PI(4,5)P_2_, is only ~ 1% of plasma membrane lipids; membranes are tightly packed with lipids and this translates to a high count of binding sites^59^.

**Figure 3:**
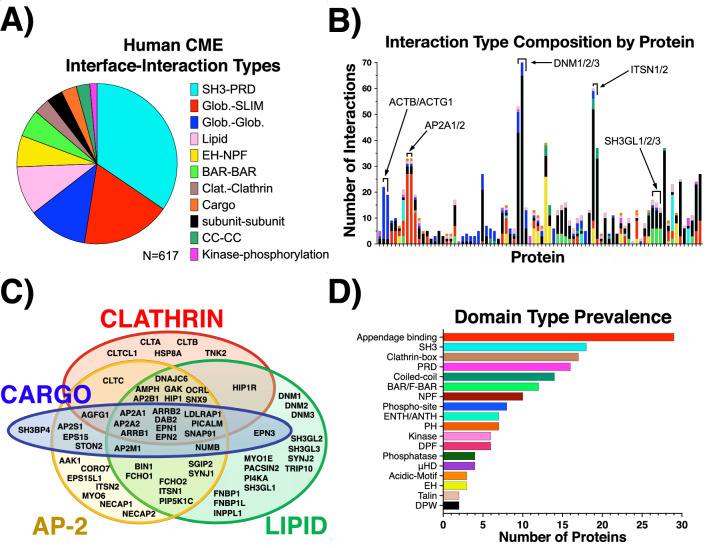
Domain types and interaction pairings in the network are dominated by binding mediated by short linear motifs (SLiMs). (**A**) The types of interaction pairings in the human CME network. PRDs, SLiMs, NPF, and Clat. categories (see Fig. 2 legend) all involve SLiMs binding to structured/globular domains. (**B**) Prevalence of domain types across all human CME proteins. Counts generated if protein has at least one domain of each type present in the CME IIN. Appendage binding, PRD, clathrin-box, NPF, DPF, acidic motifs, DPW are all SLiMs, (**C**) The majority of proteins in the network bind to at least one of the functionally critical components of the AP-2 complex, clathrin triskelion, lipid, and/or cargo. Proteins that are not present in this core set are cytoskeletal. (**D**) Interface-interaction composition profile of human CME proteins, listed alphabetically along the x-axis. Counts generated if protein contains either domain involved in interface-interaction listed. Self-interactions were counted once. Domain names are defined in Fig. 2.

The top tier of proteins, including the clathrin genes, connect to the membrane only indirectly via protein–protein interactions. The clathrin trimer is a stable complex composed of three copies of heavy chain (CLTC or CLTCL1) and three copies of the light chain (CLTA or CLTB). The Venn diagram in Fig. 3C highlights how many proteins (10) that bind to clathrin also bind the adaptor AP-2, membrane lipids, and cargo. We found only one protein, HIP1R, which binds to clathrin and lipids but not AP-2. Thus, almost all lipid binding proteins that bind clathrin also bind AP-2, but the reverse is not true. The lipid binders that are independent of AP-2 and clathrin are almost all either lipid phosphatases or BAR domain containing^62,63^ curvature sensors/inducers. The remaining components of the top-tier that are not part of our defined AP-2/clathrin/cargo core (Fig. 3C) are primarily associated with the actin cytoskeleton, which helps to bend the membrane in CME, although in mammalian cells it is not required^64^. Of the cytoskeletal components in our network, in fact, only CORO7 and MYO6 directly interacts with AP-2 or clathrin, and in our stoichiometric analysis below, we test removal of the cytoskeleton on relative abundances.

### The majority of binding interactions are mediated by weak or transient connections involving a short linear motif

We defined 11 major interface-interaction classes based on the interfaces present in our network (Fig. 3). Of the 617 interactions present in the IIN, more than half involve SLiMs (Fig. 3A). The most common specific interaction pairs are the PRDs binding to the structured SH3 domains (Fig. 3A). Most CME proteins have SLiMs that mediate interactions with AP-2, the clathrin-terminal domain, and SH3 domains (Fig. 3B). All of these binding interactions are relatively low affinity, with SH3 K_*D*_s ranging from 1 to 10 μM^65^, and clathrin-box^66^ and AP-2 appendage interactions typically in the μM regime^15,16^ (see Table S2). Selected weak interactions can be beneficial in forming stable ring assemblies^67^, and here, these low affinity interactions seem to allow for the rapid assembly and disassembly of transient complexes and clusters, as has been shown for the FCHO1-EPS15 interactions that nucleate sites of clathrin-coated pits^68–70^.

### Dynamin centers a module that is differentiable from the AP-2/clathrin/cargo module

We find the interaction composition profiles for the three dynamin proteins, DNM1/2/3, are overrepresented by SH3-PRD. For 43 of 53 total interactions mediated by DNM1 and 65 of 74 interactions by DNM2, respectively, ~ 81% and ~ 88% of their interactions are mediated exclusively by the multiple PRD motifs within dynamin, driving their high-ranking in protein connectivity (Fig. 3D). Dynamins provide a hub of a distinctive module of the CME network because they do not bind AP-2 or clathrin proteins. Their central role in membrane reshaping (e.g. via direct GTPase activity during fission)^71^, is instead augmented by their recruitment of multiple membrane remodelers like SH3-containing endophilins and formin-binding proteins, as well as cytoskeletal proteins^72^ (Fig. 3C).

The SH3-PRD domain interactions therefore act as another way to usefully partition the CME proteins by how they connect between the dynamin vs the AP-2/clathrin/cargo module (Fig. 3C). The SH3-PRD interactions are not used by AP-2 or clathrin, or to mediate cargo or lipid binding (except one protein SH3BP4). SH3-PRD interactions instead cluster with lipid binding (endophilins and formin binding proteins) and cytoskeletal proteins. Distinct classes of proteins crossover between the AP-2/clathrin/cargo module (Fig. 3C) and the SH3-PRD module. The proteins DAB2, NUMB, SYNJ1, OCRL, SGIP1, and HIP1R all connect to the AP-2/clathrin/cargo module and have PRDs. The proteins AMPH, Bin1 (amphiphysin II), SNX9 and, SGIP1 all connect to the AP-2/clathrin/cargo module and contain BAR domains and SH3 domains. Finally, intersectins (ITSN1 and ITSN2) connect to both modules and are unique in also containing EH domains that bind to several other adaptors, providing direct links to AP-2, other adaptor proteins, dynamins, and links to the cytoskeleton.

### The multiple cargo adaptor proteins each have a unique binding profile

From the network (Fig. 2A) and Fig. 1, it becomes clear that most of the 13 cargo adaptor proteins (other than AP-2) have a set of interfaces and therefore binding profiles that distinguishes them from one another. Thus, despite that nearly all of them bind to lipids, AP-2 and/or clathrin, they connect with distinct cargo and other CME proteins. Most notably, the adaptor protein DAB2 has 11 distinct protein binding partners including within the cytoskeleton, while the adaptor protein STON2 binds to AP-2, EPS15, ITSN1, and its cargo, but neither clathrin nor the plasma membrane (see also Table S3). These differences help contribute, as we see below, to distinct patterns of stoichiometry.

### Protein abundances span 6 orders of magnitude, creating variable supply and demand

**Figure 4:**
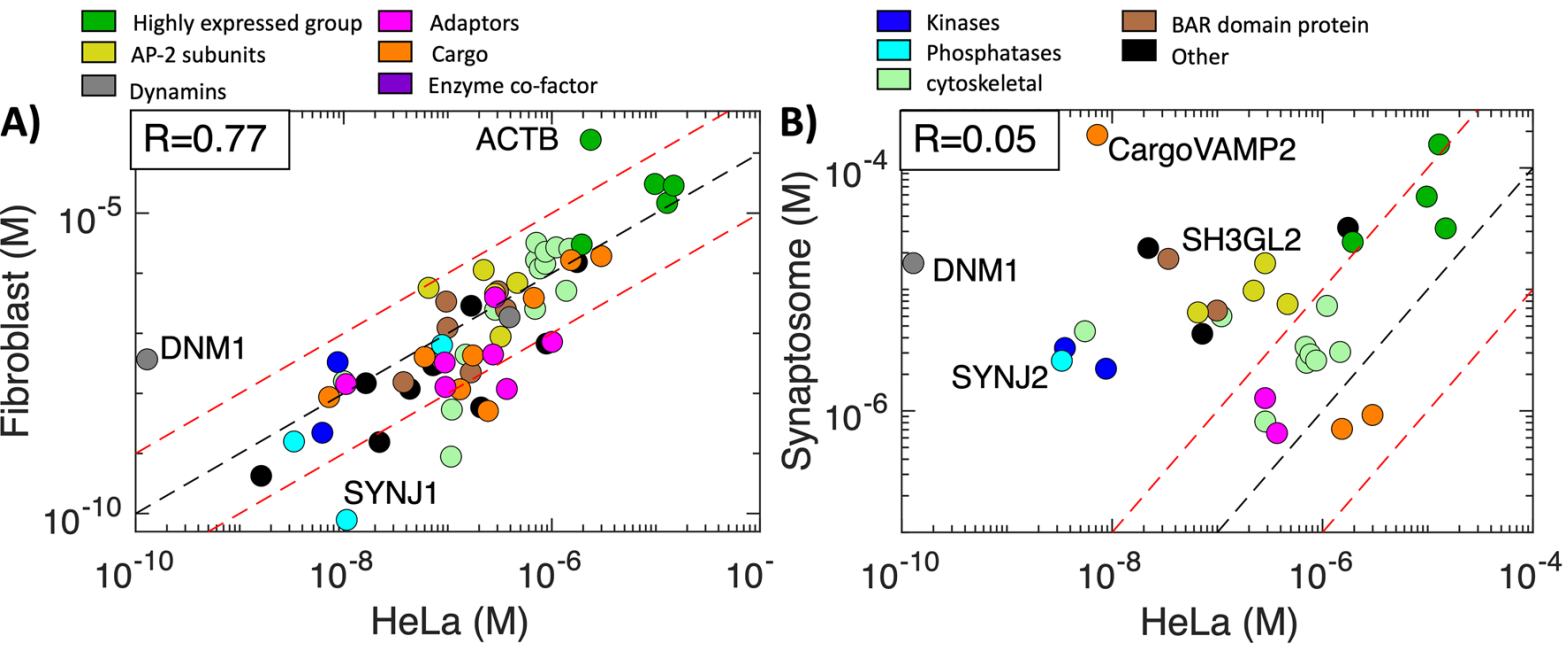
Concentrations vary by orders of magnitude across the CME proteome, with strong correlation between HeLa and Fibroblast cells. (**A**) Most proteins in Fibroblast cells are within a factor of 10 (dashed red lines) compared to HeLa cells, R = 0.77. Correlations are always applied to log10 values. (**B**) In synaptosome, although the highly expressed proteins (green: actins, clathrin heavy chain, cofilin, Hsc70) are still correlated, most proteins are significantly more abundant in synaptosomes relative to HeLa. R = 0.05. Proteins with zero copies in a cell type (e.g. SNAP91/AP180 in HeLa) or unknown copies are not shown. Importantly, although the AP-2 subunits are more abundant in synaptosomes (yellow dots), they no longer outnumber the majority of proteins, as they do in the other cell types. Outliers are labeled. DNM1 has a markedly lower concentration in HeLa than either fibroblast or synaptosome. Lipid phosphatases SYNJ1 and SYNJ2 have widely varying concentrations, with SYNJ2 and SH3GL2 (endophilin A1) much more concentrated in synaptosomes.

The observed copy numbers for the HeLa cell type, shown in Fig. 4, span multiple decades, from high pM for dynamin1 DNM1 to > 10 μM for cofilin CFL1, with the central lipids PI(4,5)P_2_ and PI/PS reaching even higher concentrations (Fig. S2). Although lipids and transmembrane cargo are restricted to the 2D membrane, we can define their concentration via copies/cell volume. Importantly, all of the comparisons of copy numbers and stoichiometries are done on a log10 basis, to meaningfully compare trends across all copy numbers, rather than be dominated by discrepancies in abundant proteins. Therefore, noise in copy numbers, which is expected in experimental measurements^1^, has a marginal influence on our statistics. Notable deviations in abundance and stoichiometry occur at orders of magnitude or at least 50% variation, typically outside of expected noise, and thus precise copy numbers are not necessary for these calculations.

### Abundances are highly correlated from HeLa to fibroblast, but not synaptosomes

From HeLa to fibroblast, protein concentrations have significant correlation of R = 0.77 (Fig. 4A), close to the correlation across two HeLa studies (R = 0.83 Fig. S3). Most concentrations are within a factor of 10 of the other cell type, with outliers including the highly expressed ACTB, which increases from 2.4 to 166 μM in fibroblast, and SYNJ1 and DNM1, which have very low abundances in fibroblasts and HeLa cells, respectively. In contrast, the concentrations in the synaptosome have minimal correlation with HeLa, with R = 0.05. The strongest correlation remains in the highly abundant proteins (CLTC/clathrin heavy chain, actins:ACTB and ACTG1, CFL1/cofilin, HSPA8/HSC70), which are quite similar in concentration. However, most proteins have much higher concentrations in the synaptosome compared to HeLa (Fig. 4B). This is likely in part due to challenges in measuring very low copy numbers: the synaptosome is a small compartment with a volume ~ 6000 times smaller than the HeLa cell^5^, so a single copy of a protein is already at 7 nM concentration, vs 1 pM in a HeLa. The sharp contraction in overall abundances in the synaptosomes means that for highly connected proteins such as AP-2 that have dozens of binding partners, they are far less likely to be more concentrated than their partners. For AP-2, its partners in HeLa cells span a factor of 1000 in abundance relative to AP-2 subunits, but are all within a factor of 10 in the synaptosome. As a result, AP-2 subunits are significantly less concentrated relative to many of their partners in the synaptosome, which influences its stoichiometry below. A few proteins in each cell type had a known abundance of zero copies (e.g. WAS, SNAP91/AP180, SH3GL3) and were thus excluded from the calculations (Table S1).

However, we find that despite differences in abundances of individual proteins, the protein classes show broadly similar trends across cell types (Fig. S4). Enzymes have the lowest mean concentrations, and cytoskeletal components have high mean concentrations. We found that the subunits of obligate multi-protein complexes (the 7 subunit ARP2/3, the 4 subunit AP-2, and the two-component clathrin trimer) indeed had the lowest variation (Fig. S4), as expected from stoichiometric demands and as is observed experimentally^45,46^.

### Stoichiometry defined only by nearest neighbors overestimates demand on individual proteins

**Figure 5:**
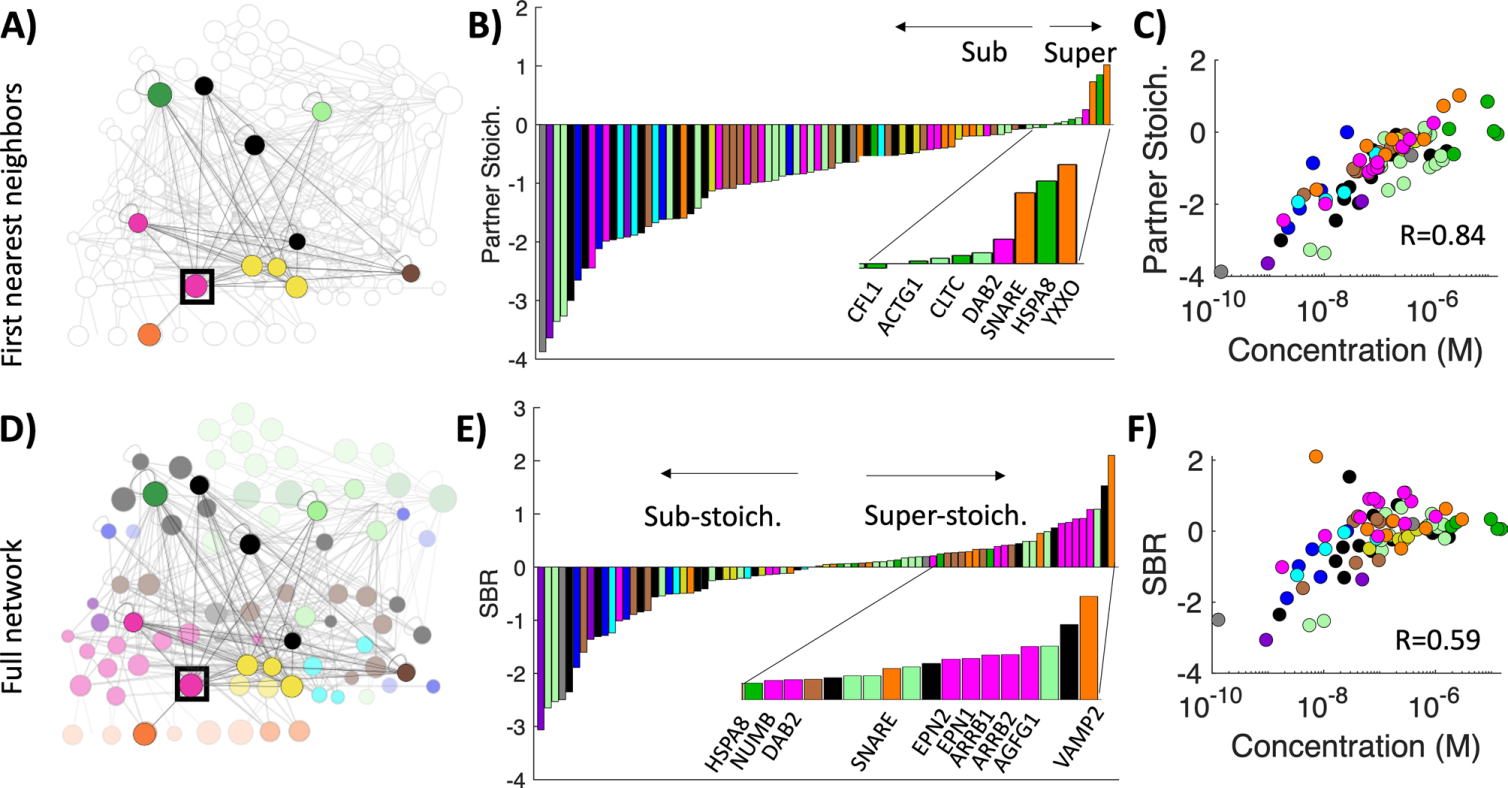
Stoichiometry of each protein to its direct binding partners, or to the full network, shows distinct patterns for highly abundant proteins. (**A**) A simple model of balance compares a protein only to its immediate binding partners or nearest neighbors, shown here for cargo adaptor DAB2. Node size is scaled to log10(abundance). (**B**) Competition for single interfaces results in most proteins being sub-stoichiometric, or without sufficient supply to meet partner demands. Stoichiometry is on a log10 scale, each integer thus indicating orders-of-magnitude differences between experimentally observed copies (here for HeLa cells^2,3^) and balanced copies. (**C**) Because only nearest neighbors are considered, partner stoichiometry correlates strongly with concentration, with abundant proteins most likely super-stoichiometric. (**D**) With the stoichiometric balance method, not only first neighbors (dark nodes) but second (faded) and beyond (more faded) neighbors contribute to overall stoichiometry through their competition. (**E**) With the SBR, many sub-stoichiometric proteins are similar to the simpler balance method. However, super-stoichiometric proteins now include most of the cargo adaptor proteins (pink), which are all competing with one another to bind AP-2 and clathrin. (**F**) The SBR has weaker correlation with concentration, as proteins with lower abundance are also super-stoichiometric. The cargo VAMP2 has low abundance but is super-stoichiometric, due to competing with the abundant class of SNARE_class proteins for internalization.

The most straightforward way to measure stoichiometry is to compare a protein’s copies to its copies of binding partners, while accounting for each distinct interface. Stoichiometry can thus be measured for each interface on a protein, and in Fig. 5 we then average over all interfaces to report a single number per protein. This partner stoichiometry (SP) for a protein *p* is given by:

1 $${\mathrm{SP}}_{p}={\mathrm{log}}_{10} \left(\frac{{C}_{obs}\left(p\right)}{\frac{1}{{N}_{iface}(p)}{\sum }_{i=1}^{{N}_{iface}(p)}{\sum }_{k=1}^{NP\left(i\right)}{C}_{obs}\left(k\right)}\right)$$

where ${C}_{obs}$ are observed copy numbers taken from experimental literature^2,3^, ${N}_{iface}$ is number of interfaces and $NP\left(i\right)$ are the number of binding partners of interface *i*. The vast majority of proteins based on this metric are sub-stoichiometric, or of insufficient supply (Fig. 5B). This metric is strongly correlated with abundance, with an R = 0.84 (Fig. 5C), as most proteins have more than one partner but the variation (~ 1–50 partners) is much less than the variability in concentrations, meaning proteins of below average abundance are much more likely to be outnumbered by their partners. The chaperone HSPA8/HSC70 and a few cargo classes (SNARE_class, YXXØ_class) are much more abundant than are demanded by the CME network. Clathrin heavy chain (CLTC) is also super-stoichiometric, although we note that its terminal domain interface that is specific for the clathrin box interface (providing a primary link to the adaptor network) is actually sub-stoichiometric. Clathrin is on average in excess supply because in addition to this interface, it contains multiple additional interfaces (Table S2) that are super-abundant. We note that we include all proteins and cargo classes when calculating stoichiometries, except for proteins/cargo that have known copy numbers of zero in a given cell (e.g. SNAP91/AP180 in HeLa and fibroblast). We exclude lipids because their very high relative abundances (from ~ 0.1 to 10% of plasma membrane lipids, compiled here^59^) dilute the correlations observed between proteins. Additionally, lipids like phosphatidylserine do not bind proteins at a 1:1 stoichiometry, and they recruit many other proteins outside of CME. A significant limitation of this metric is that it assumes all partners are exclusively available to bind only protein *p*.

### Network stoichiometric balance identifies competition between adaptor proteins for binding clathrin and AP-2

To expand our measure of stoichiometry to account for the full network of interactions simultaneously, we define a set of balanced copy numbers for each protein in the network that can be compared to its observed copy numbers. Our model of stoichiometric balance defines balanced copy numbers as matching up each copy of an interface to one of its partners, where each partner is assumed to compete with equal strength^39^. The model thus identifies where regions of the network produce large imbalances in stoichiometry of binding partners, while accounting for shared and distinct interfaces on each protein. We constrain the balanced copy number solutions to be as close as possible to the observed, real copies for a protein. For multi-interface proteins, we further constrain each domain within the proteins to have similar copy numbers (i.e. a specific protein should not have 1 million copies of one domain, and only 10 of another); we allow variance due to known fluctuations in protein copy numbers^1^. The model is solved using nonlinear optimization performed by the SBOPN method (Stoichiometric Balance Optimized for Protein Networks), which takes < 1 s (see “Methods”) ^39^. A toy network example is provided in Fig. S5. The balanced copy numbers for a protein *p* are averaged over the balanced copies for each interface, ${C}_{balanced}\left(p\right)=\frac{1}{{N}_{iface}(p)}{\sum }_{i=1}^{{N}_{iface}(p)}{C}_{balanced}(i)$. We define the stoichiometric balance ratio, or SBR, of a protein *p* as the ratio between observed (*C*_obs_) and balanced (*C*_balanced_) copy numbers, which determines whether a protein is sub- or super-stoichiometric.

2 $${\mathrm{SBR}}_{p}={\mathrm{log}}_{10} \left(\frac{{C}_{obs}\left(p\right)}{{C}_{balanced}\left(p\right)}\right)$$

Perfectly stoichiometric proteins thus have an SBR = 0, regardless of whether they have high or low abundance. Proteins that have too few observed copies, or are in demand, are sub-stoichiometric (SBR < 0), and proteins with too many observed copies, or have excess supply, are super-stoichiometric (SBR > 0). The SBR indicates the order-of-magnitude deviations, for example, an SBR = 4 means the observed copies are 10^4^ times more abundant than required to balance its partners.

From Fig. 5E, we can see that the SBR metric shows significant differences in super-stoichiometric proteins relative to the simple partner stoichiometry metric (Fig. 5B). The most noticeable trend is the number of adaptor proteins (pink) that are now super-stoichiometric, whereas based on a partner metric, only DAB2 was in excess supply. The origin of this is the extensive competition of most of these adaptor proteins to bind one interface on clathrin heavy chain/CLTC, and to bind the appendage domain on the AP-2 β subunit/AP2B1. The majority of adaptors also outnumber the cargo (orange) they select for (Table S4), with only the YXXØ_class (which includes the abundant transferrin receptor) and the SNARE_class of cargo in excess. If we remove all cargo proteins from the network and re-evaluate the SBR, the overall distribution is similar but the adaptor proteins exhibit even larger scales of imbalances in stoichiometry (Fig. S7). The adaptor proteins STON2 and EPS15 are sub-stoichiometric because they do not compete to bind the clathrin heavy chain/CLTC. This illustrates how differences in network connectivity drive altered demand even when abundances, such as between EPS15 and EPN2, are almost identical.

We note that the SBR shown in Fig. 5E is based on an average over all interfaces on a protein, and each individual interface can have an SBR that differs due to variations in interface copy numbers. While we discuss specific examples further below, the proteins with the largest coefficient of variation (CV) of balanced copies across interfaces include the highly super-stoichiometric adaptor proteins, and most of the enzymes (Fig. S6). In these cases, the proteins each have interfaces that compete to bind the AP-2 adaptor proteins and are assigned low balanced copies, but also contain other protein interaction domains that can accommodate much higher copies. In contrast, highly abundant proteins have lower CVs, as do the dynamins and BAR domain proteins. Overall, the proteins with high variability typically contain two types of interfaces: an interface that binds to a hub and is thus in competition with many other proteins, and an interface that binds to another domain exclusively, and thus must match up abundances with that partner protein, providing more limited flexibility on stoichiometry.

### Stoichiometric Balance Ratio correlates with abundance

We find that the correlation of protein abundance with SBR is significant, with R = 0.59 (Fig. 5F), although less so than the partner stoichiometry. The drop in correlation is driven in part by the proteins identified in our analysis as being super-stoichiometric, despite having relatively low abundance. A significant outlier is VAMP2, which has very low but measureable abundance (0.007 μM). Although VAMP2 is a SNARE protein, it was separated from the SNARE_class due to distinct specificity for selection in neuronal cells by the adaptor SNAP91/AP180, which has zero copies in HeLa. Thus, VAMP2 is super-stoichiometric in HeLa cells because it effectively groups with the highly abundant SNARE_class (1.5 μM), leading both to be super-stoichiometric relative to their insufficiently abundant adaptor PICALM (0.28 μM). Due to its links to both the AP-2 and clathrin proteins, PICALM is itself also super-stoichiometric, illustrating how the SBR is influenced by competing demands on binding. If PICALM only bound to its SNARE_class cargo, it would classify as sub-stoichiometric. Despite these outliers, many of the lowest abundance proteins are indeed sub-stoichiometric, notably all of the kinases and phosphatases in the network, all of which exhibit concentrations < 100 nM (Fig. 5E and Fig. S4). This same trend with kinases/phosphatases being sub-stoichiometric was observed in yeast^39^. Because transient enzyme interactions result in long-lived chemical modifications to substrates, a one-to-one stoichiometry of enzymes with targets would seem unnecessary. The low abundance co-factors auxilin/DNAJC6 and GAK, which are needed for the disassembly ATPase HSC70/HSPA8, are also both sub-stoichiometric, particularly auxilin/DNAJC6 which is present at only a few hundred copies. Their highly mismatched stoichiometry is exacerbated by the fact that they both bind to the highly abundant proteins HSC70/HSPA8 and clathrin heavy chain/CLTC.

### Stoichiometry is remarkably similar across the CME network with the cytoskeletal proteins removed

Because the cytoskeletal proteins are not strictly essential for mammalian CME^64^, and because they are involved in many other cellular pathways, we reperformed the SBR after removing them from the network. Despite representing ~ 25% of our cytosolic proteome (22 proteins), the stoichiometry is remarkably similar (Fig. S8). This result highlights how the cytoskeletal components, as noted above, lack strong connections to the central clathrin and AP-2 genes, with only two direct links to them. After cytoskeleton removal, the stoichiometry of the remaining proteins, especially the adaptor proteins and their cargo, continues to be dominated by the extensive competition to bind clathrin heavy chain/CLTC and AP2B1. The one noticeable change is that the phosphatases OCRL and SYNJ1, neither of which binds CLTC or AP2B1, are now super-stoichiometric. This flip occurred due to primarily indirect links to the binding network of their PRDs, as some cytoskeletal proteins and many of their direct partners contain multiple SH3 domains.

### Differences in stoichiometry across cell types largely track differences in concentration

Performing the same SBR analysis in the fibroblast and synaptosomes, we recover several of the same trends as in the HeLa cells (Figs. S9 and S10). Both the partner stoichiometry and the SBR values correlate with protein abundance, and in all cell types, the SBR has only a very weak correlation with network connectivity (Fig. S11). This weak correlation in all cell types is likely because protein abundances span orders of magnitude, having a wide impact on the overall SBR distribution, whereas protein connectivity only spans two orders of magnitude (1–~ 100). Abundance also does not correlate with connectivity (Fig. S11) resulting in conflicting pressures on the stoichiometry of proteins such as DNM1 with high connectivity but very low abundance.

**Figure 6:**
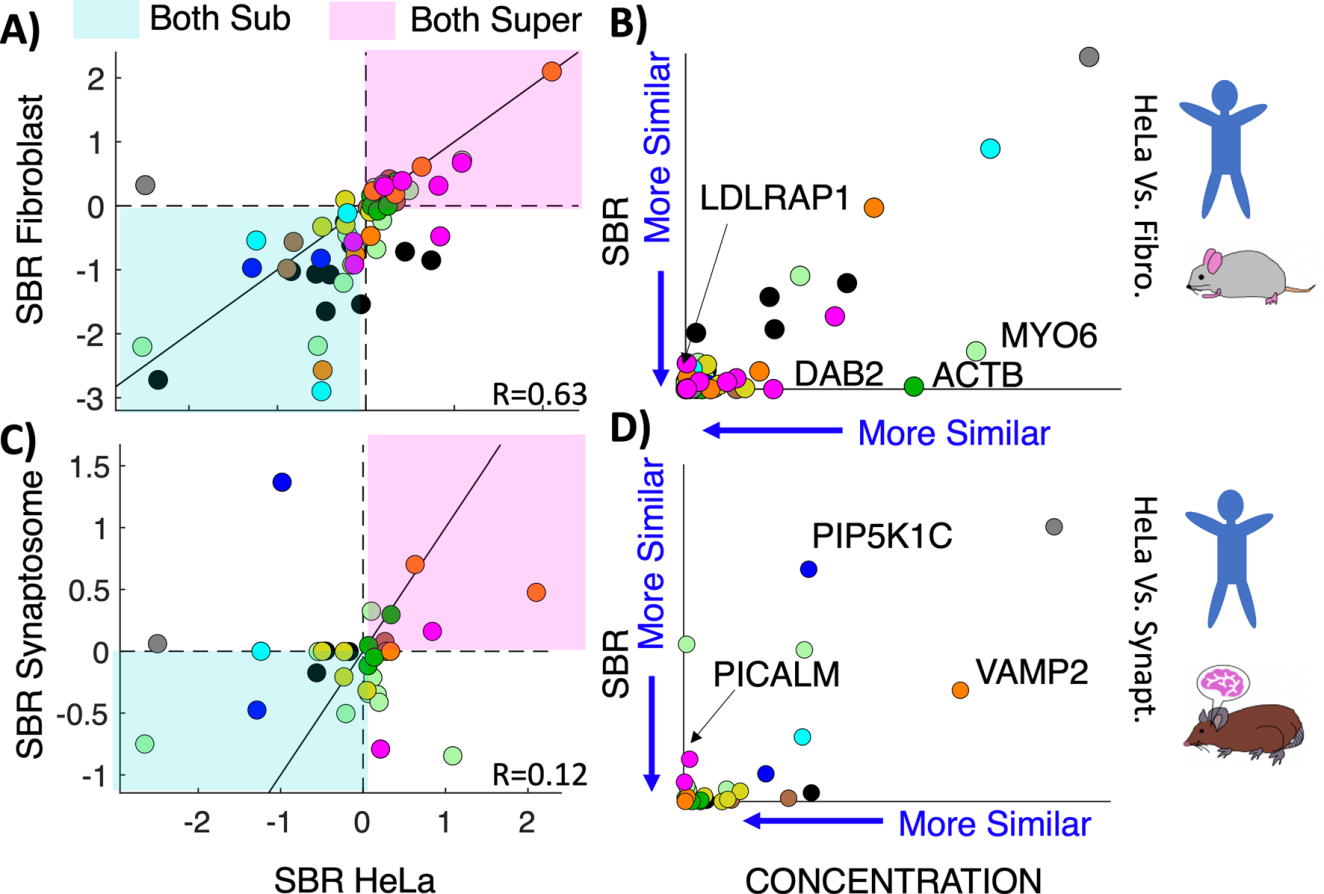
Stoichiometry is similar between HeLa and fibroblasts, with deviations largely correlating with large differences in concentration across cell types. (**A**) SBR values for fibroblast cells vs HeLa show significant correlation, with R = 0.63. (**B**) The SBR ratios between the cell types are more similar as the concentrations between the cell types are more similar, as expected and emphasized by all the points near the origin. Moving out along the diagonal are proteins that have increasingly distinct concentrations, typically driving large deviations in SBR values. Outliers are therefore along the axes, where MYO6 and DAB2 have relatively distinct abundances between the cell types, but very similar SBRs. In this case, these interacting proteins both have correlated decreases in abundance, contributing to their similar stoichiometry. LDLRAP1 has almost identical concentration in both cell types, but distinct stoichiometry, due to the drop in its competitor DAB2’s abundance. (**C**) The SBR is quite distinct in the synaptosome relative to HeLa, with an R = 0.12. (**D**) The variation in SBR is again largely resulting from large variations in concentration between the cell types. PICALM has very similar abundance in both cell types, but its cargo, including VAMP2, has a dramatic increase in abundance, contributing to a distinctive stoichiometry.

Directly comparing the SBR values across the cell types, we see in Fig. 6 that the correlation is high for HeLa vs fibroblast (R = 0.63), but weak for HeLa vs synaptosome (R = 0.12). Thus, the SBR distributions largely track with correlations in protein concentrations between the cell types, which are high for fibroblast and low for synaptosome (Fig. 4). We find that many of the large differences in stoichiometry occur for proteins such as DNM1 and SYNJ1/SYNJ2 that have widely varying concentrations from one cell type to the other (Fig. 6B,D). However, we also observe cases where concentrations are quite distinct, but the stoichiometries are almost unchanged. The proteins DAB2 and MYO6 have significant differences in concentration from HeLa to fibroblast cells, but very similar stoichiometries. In this case, these interacting partners have correlated drops in abundance, helping to contribute to similar overall SBR values as observed in HeLa cells. In contrast, the adaptor protein LDLRAP1 has almost identical concentrations in HeLa and fibroblast, but its stoichiometry has changed significantly; this is due to the drop in its competitor DAB2’s abundance by 14-fold. A notable outlier for the synaptosome is PIP5K1C, a lipid kinase that binds to AP2B1 and is super-stoichiometric in synaptosomes, but sub-stoichiometric in both HeLa and Fibroblast (Fig. 6D). Here, the abundance of PIP5K1C has increased by a factor of over 900 in the synaptosome relative to HeLa, making it far more abundant than necessary for its sole protein partner of AP2B1. The dramatic increase in PIP5K1C could be driven by its role in converting the lipid PI(4)P into PI(4,5)P_2_, an action that is reversed by the phosphatase SYNJ2. SYNJ2 is ~ 800 times more abundant in the synaptosome than in HeLa, and we speculate that maintaining the lipid homeostasis would demand similar increases in PIP5K1C.

Another notable different in the synaptosome occurs in the adaptor and cargo proteins that select for SNARE_class necessary for synaptic vesicle turnover. VAMP2 has a very high concentration of 187 μM, which exceeds any other cargo in any cell type, whereas it is present at ~ 0.008 μM in HeLa and fibroblast. In contrast, the SNARE_class and the adaptor protein PICALM (which selects for the SNARE_class and VAMP2) have very similar abundances in both HeLa and synaptosomes. Thus, because of the dramatic increase in the cargo VAMP2, the SBR of PICALM has changed significantly (Fig. 6D). An additional adaptor protein SNAP91/AP180 partially compensates for the cargo increase, as it is not present in HeLa or fibroblast, also selects for both VAMP2 and SNARE_class, and is now the most concentrated adaptor protein in synaptosomes, at 26 μM. However, it is still greatly outnumbered by the VAMP2 copies. We note that because we use the total abundances in each cell type for our stoichiometric comparison, we do not account for whether specific molecules such as VAMP2 are primarily found within synaptic vesicles rather than at the plasma membrane, which could alter their abundances by factors of ~ 10–100. We do not consider this here because it would require estimating new copy numbers for all the CME proteins, based on their engagement in distinct cellular pathways. Also, this redefinition of copy numbers would impact our relative stoichiometries only if some proteins had significant (orders-of-magnitude) depletion via other pathways while others did not, as the stoichiometry is dominated by changes over three to six orders-of-magnitude.

### Perturbing the network by protein removal reveals global and local regulators of stoichiometry

**Figure 7:**
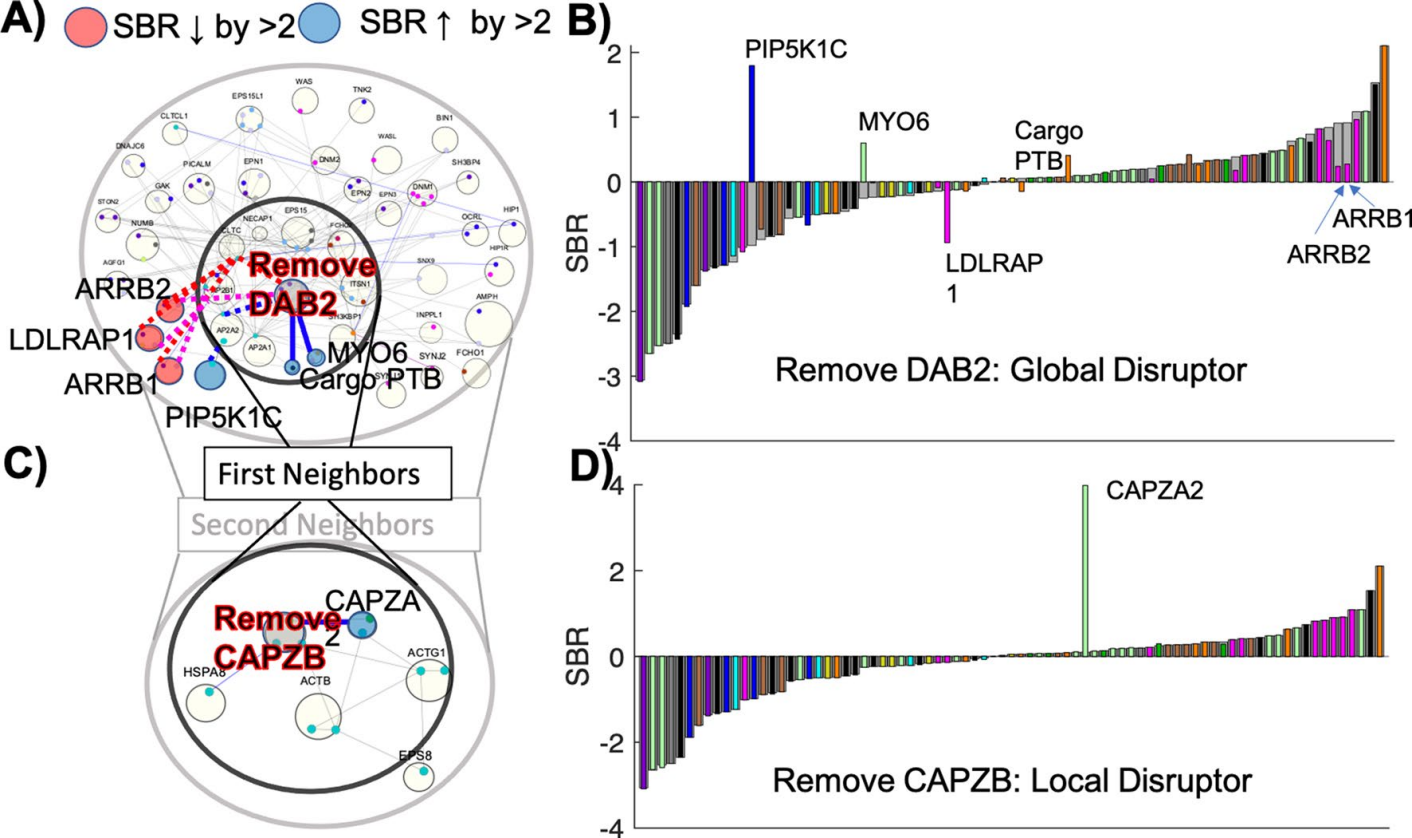
Removal of individual proteins from the network reveals their role as local or global controls of stoichiometry. (**A**) Removal of the multi-interface and highly connected DAB2 results in changes to stoichiometry to not only direct neighbors, but second neighbors (via interfaces) that compete with DAB2 to bind the same interfaces on AP2B1 and CLTC (red and pink dashed lines). (**B**) SBR of the HeLa network is shown ordered in gray, with the response following DAB2 removal in color bars. Proteins whose SBR changes by more than 2 are labeled in both (**A**) and (**B**). (**C**) Removal of CAPZB impacts stoichiometry of only its nearest neighbor CAPZA2. Partners of its immediate binding interfaces are shown. (**D**) SBR is essentially unchanged following CAPZB removal except for CAPZA2.

By removing each protein from the network, we can clearly see how a single protein can cause pronounced global or local effects on the SBR distribution of the full protein network, or have no detectable impact. A most striking result is the response to DAB2 removal (Fig. 7A,B) which shifts the stoichiometry of several other adaptor proteins that are not its direct binding partners. The adaptor proteins ARRB1, ARRB2, and LDLRAP1 all compete with DAB2 to bind clathrin and AP-2. Because DAB2 is the most highly expressed cargo adaptor in HeLa cells at ~ 1 μM, when it is removed, there are many more interfaces available on AP-2 and clathrin to form complexes with these alternate adaptors, driving their SBR values closer to 0. DAB2 removal also impacts its direct binding partners, causing the relatively peripheral Myosin 6 (MYO6) to switch from being sub- to super-stoichiometric as it now has far fewer binding partners within the CME network. Without DAB2, there would naturally be a reduction in uptake of its target cargo, the LDL receptor, as is observed experimentally^73^. With more space for other adaptor proteins to be internalized with their corresponding cargo, DAB2 removal would therefore correlate with increased uptake of distinct target cargo, and indeed the amount of transferrin receptor on the plasma membrane was reduced, indicating increased uptake of distinct cargo^73^. We note that this compensation in which cargo is taken up is not found with all experimental knockdowns of adaptor proteins^74^, because the adaptors also can contribute to efficient clathrin coat assembly. DAB2 seems to operate more independently of AP-2 to coordinate uptake of its own cargo, as it does not need AP-2 binding to internalize LDL receptors^73^.

Unlike the more global SBR shift accompanying DAB2 removal, a strong local coupling emerges for F-actin capping protein subunit b (CAPZB) and F-actin capping protein subunit a (CAPZA2), which have similar copy numbers to one another (Fig. 7C,D). Both proteins have low connectivity in the network (Fig. 2) but contain interfaces to the highly abundant and connected actin interfaces. Importantly, their networks are not identical: CAPZB also binds the highly abundant HSPA8/HSC70. After applying the SBOPN algorithm, CAPZA2 and CAPZB copy numbers are quite irregular from one interface to the next. As a result, when CAPZB is removed, the SBR of CAPZA2 changes dramatically, with no other proteins affected.

### Removal of hub proteins and highly abundant proteins is more disruptive to the network stoichiometry

**Figure 8:**
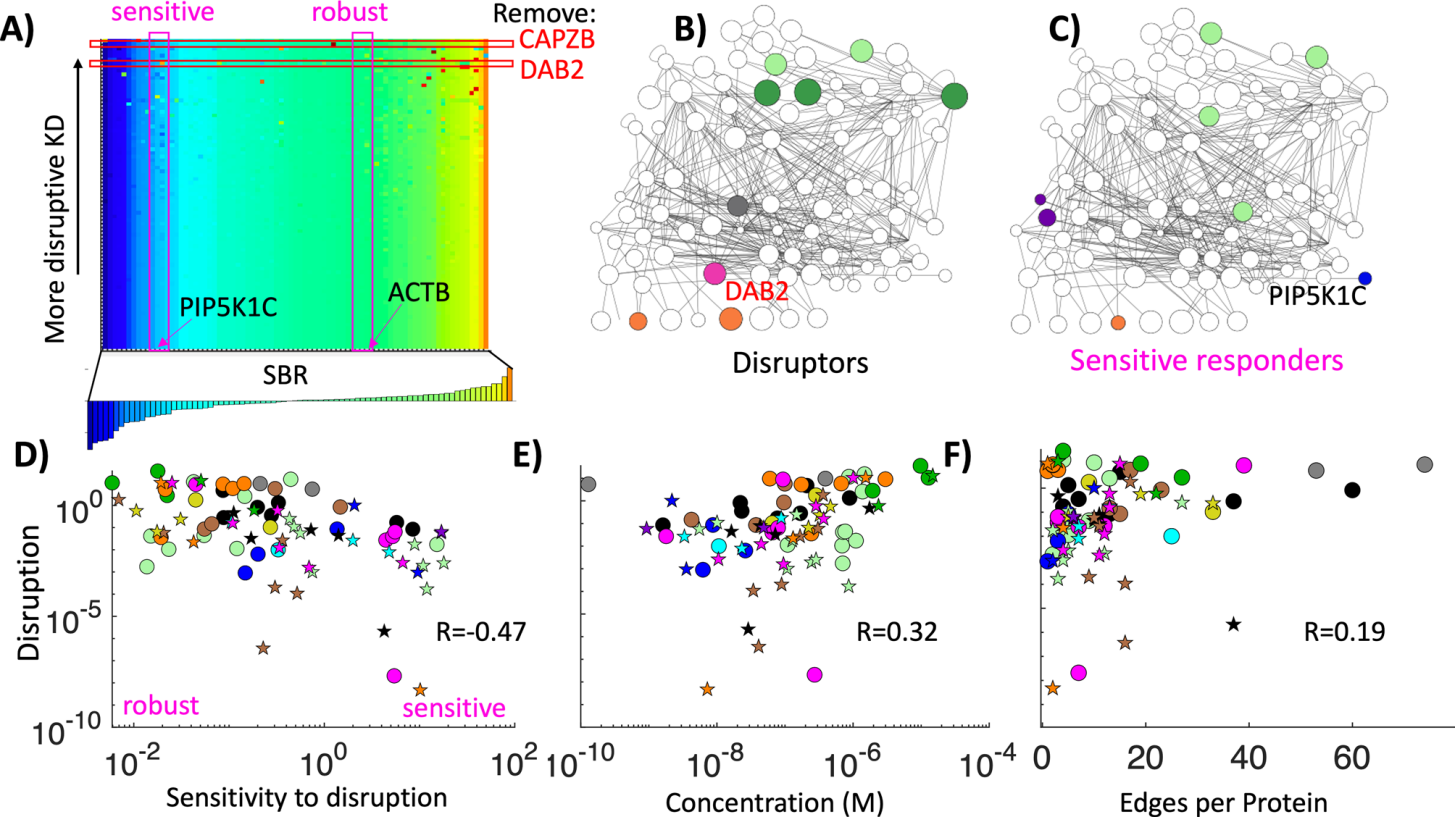
Systematic removal of each node from the network shows that highly abundant proteins control and disrupt stoichiometry upon removal most strongly. (**A**) The SBR distribution of the HeLa network (bottom inset) can change as a single node is removed (“KD: knockdowns”), where the color bar indicates the SBR value, with blue being most sub-stoichiometric and red being most super-stoichiometric. Each column thus records SBR of the same protein in the network. Each row follows a distinct knockdown, with rows at the top indicating the most disruptive removals (e.g. DAB2 and CAPZB). Some proteins are quite sensitive to these network perturbations with widely varying SBR values (magenta column: sensitive e.g. PIP5K1C), while others are robust to all single node perturbations (e.g. ACTB). (**B**) Most disruptive nodes are the most abundant proteins (dark green), other highly abundant and connected proteins (pink and gray), and a few cargo proteins that compete for binding. (**C**) Proteins most sensitive to network perturbations are more peripheral, including PIP5K1C, the co-factors that bind CLTC and HSPA8/HSC70, and several cytoskeletal proteins with few links. (**D**) Disruptive proteins are typically robust to network perturbations, producing an anti-correlation between disruption distance (Eq. 3) and sensitivity distance (Eq. 4). Proteins that have the largest impact on nearest neighbors are shown as circles, and on second nearest neighbors as stars, showing how both types of perturbations are common throughout the network. (**E**) Disruption is correlated with concentration, with R = 0.32. Notably, proteins with concentration > 2 μM are all strong disruptors. (**F**) Disruption has weaker correlation with edges, where only proteins that have > 50 edges are reliably disruptive to the network stoichiometry upon removal. Both disruption and sensitivity have almost the same values if they are evaluated on a per interface level first, before assigning a single SBR value to each protein, with a correlation of R = 0.98 between both values.

After knocking down each protein from the network, we can rank which proteins have the biggest impact on the SBR distribution in the network (Fig. 8A). We quantify the impact by the Euclidean squared distance between the SBRs across the network before (${\mathrm{SBR}}_{p})$ and after a protein $\mathrm{\alpha }$ is removed,

3 $$\mathrm{Disruption}(\mathrm{\alpha })=\sum_{p=1}^{N}{({\mathrm{SBR}}_{p}^{\mathrm{\alpha }}-{\mathrm{SBR}}_{p})}^{2}$$

where *N* is the number of proteins in both networks. Several proteins have virtually no impact on the network SBR when they are removed. When we sort the proteins that have the biggest impact, we find that the most abundant proteins (Fig. 8B) and the most highly connected proteins are all highly disruptive upon removal (Fig. 8E,F). In other words, the disruption index has a positive correlation with protein abundance, and a positive correlation with protein connectivity (Fig. 8E,F). The correlation is significant but not strong, in part because connectivity and copy numbers are not correlated, and because there are a number of coupled nodes that need not have high copies or connectivity to strongly disrupt the SBR of one protein, including CAPZB and CAPZA2 (Fig. 7), and the components ACTR3 and ARPC3 of the ARP2/3 complex. The same trends are observed across all three cell types, where HSPA8/HSC70 is always disruptive upon removal, as are at least one of the more abundant adaptor proteins and cargos, such as DAB2 (Hela and fibroblast) and SNAP91/AP180 (synaptosome) (Fig. S12). The same trend is also observed when the cytoskeletal proteins are removed from the network prior to perturbation analysis, with DAB2 and HSPA8/HSC70 being most disruptive (Fig. S8).

### Proteins with stoichiometry that is sensitive to removal of others have low network connectivity

As the inverse of disruption, we can quantify which proteins have an SBR that is sensitive to perturbations to the network. The sensitivity can be measured much the same way as the disruption index, now comparing the SBR value for a single protein $\alpha$ as it changes across each protein removal

4 $$\mathrm{Sensitivity}\left(\alpha \right)=\sum_{k=1}^{{N}_{KD}}{({\mathrm{SBR}}_{\alpha }^{\mathrm{k}}-{\mathrm{SBR}}_{\alpha })}^{2}$$

where *N*_KD_ are the number of proteins that are removed (KD: knocked-down), ${\mathrm{SBR}}_{\alpha }$ is the value in the original network for protein $\alpha$, and ${\mathrm{SBR}}_{\alpha }^{\mathrm{k}}$ is the value when protein *k* is removed. Several proteins have robust SBR values that barely change at all across any single node removal (Fig. 8A). These robust proteins tend to be the ones that are most disruptive upon removal (Fig. 8B), producing a significant anti-correlation of R = − 0.47 between disruption index and sensitivity (Fig. 8D), with similar values observed in fibroblast and synaptosomes (R = − 0.41, R = − 0.41). For example, when the disruptor protein DAB2 is removed, it induces a broad change in the stoichiometry of other proteins, but its SBR is insensitive to removal of other proteins. On the other end is LDLRAP1, whose SBR is sensitive to removal of an array of other proteins in the network, but whose own removal hardly disrupts the SBR distribution at all. This result highlights how despite their shared function as cargo adaptor proteins for the same cargo (LDL receptor), DAB2 and LDLRAP1 have dramatically distinct impact on network stoichiometry due to their distinctive binding networks and abundance. Sensitive proteins typically have fewer connections in the network such as PIP5K1C (Fig. 8C), and as peripheral proteins their SBR is more controlled by their more highly connected partners. The co-factors DNAJC6/auxilin and GAK (Fig. 8C purple nodes) are both quite sensitive because they have low abundance and connectivity, but bind to the highly abundant and disruptive proteins HSPA8/HSC70 and CLTC/clathrin heavy chain. The peripheral actin binder DBNL is highly sensitive across all cell types, as despite having moderate abundance (0.25–0.8 μM), it has only 3 connections to the highly connected proteins ACTB, ACTG1, and DNM1. Not only does removal of its immediate partners alters its SBR, but several actin or DNM1 partners can impact its stoichiometry. Sensitive proteins also typically have larger coefficients of variation in balanced copies optimized for each interface, including DBNL, DNAJC6/auxilin and GAK (Fig. S6). As a result, with removal of partners that target their distinct interfaces, the SBR can respond significantly.

## Discussion

The construction and analysis here of the mammalian interface-resolved CME network integrates structural data, binding interactions, and protein abundances to reveal patterns of stoichiometry that reflect competition and cooperation for binding not visible from a protein–protein network. Our network captures the extensive competition for specific interfaces on AP-2 and clathrin, while also showing the numerous additional interfaces every protein uses to form distinctive binding networks through permutations of similar domain types. Many of these variations would not be visible without extending the network out from a smaller core subset of clathrin and adaptor proteins to include many proteins that assist in membrane remodeling, such as BAR domain proteins. While many protein functional classes show trends in abundance across all cell types, with the SBR analysis we characterize each individual protein, identifying stoichiometric imbalances in binding partners that extends beyond a simple neighbor analysis to capture competition for binding across the network. Clathrin is highly abundant, but it is outnumbered by all the adaptor proteins that compete to bind an interface on its terminal domain. However, because clathrin has additional interfaces that are super-abundant, and because its adaptor binding partners are adjusted for whether they are sub- or super-stoichiometric due to their other binding partners, the balanced copies of clathrin are still in overall excess supply. One of our more surprising findings is how cargo adaptor protein that are quite similar functionally like DAB2 and LDLRAP1/ARH, with key commonalities linking to the membrane, clathrin, and AP-2, can nonetheless display varying SBR values and disruptiveness upon removal. Our analysis demonstrates how DAB2 plays a dominant role impacting stoichiometry in HeLa due to its high abundance and connectivity, but that in synaptosome, the adaptors PICALM and SNAP91/AP180 that select for the highly abundant SNARE_class protein cargo are more impactful. By integrating detailed structural networks and abundances, these distinct features of individual adaptor proteins or cargo proteins emerge by comparing stoichiometry of binding using quantitative and objective metrics such as the SBR that are intuitive even though they require computer optimization.

Our analysis predicts which proteins are most disruptive and most sensitive upon ‘knockdowns’ or removal of other components of the CME network; above we made a specific comparison to experiment upon removal of DAB2 as it impacts cargo uptake^73^. More broadly, we think the predictions from our analysis could be productively combined with proteomic analyses^49^ performed after protein knockdowns, as these could more globally report on how the composition of vesicles^49^ would change with each protein removal. Our analysis would predict that larger shifts in composition would follow removal of ‘disruptor’ proteins like DAB2 and other highly-connected proteins like the AP-2 subunits^54^. Further, we would predict that the behavior of sensitive proteins, such as the disassembly co-factors DNAJC6 and GAK, would frequently shift following knockdown of several other proteins. Tracking uptake of many distinct types of cargo receptors at once following knockdowns would also be a valuable comparison for our model predictions, as we can assess the response of not just immediate binding partners, but indirect interactors.

A limitation with the stoichiometric balance measured here is that SBR values can vary depending on the network structure, and the calculation relies on having experimentally measured copy numbers of proteins. The stoichiometric imbalances are also only meant to be interpreted in light of the specific network. In the fibroblast and synaptosome cell types, multiple proteins had unknown abundances, and optimizing the network stoichiometry around these components allows them to at times act as ‘buffers’, absorbing large imbalances in copy numbers. This becomes quite visible when these buffer proteins are removed and cause large disruptions to the network stoichiometry. However, this also means that the stoichiometry for any individual protein can be re-evaluated as its network of binding partners is perturbed, indicating how strongly coupled the protein is to any specific network. Without the cytoskeletal proteins, which are relatively weakly linked to the central clathrin/adaptor core of the network, the SBR and its response to perturbations are remarkably similar to the full network. Because the SBR calculations are efficient (< 1 s), systematic perturbations are straightforward, and we found that the most abundant proteins are the most disruptive to stoichiometry, more so than the most highly connected proteins. These abundant proteins are also insensitive to perturbations; most core stoichiometric relationships in the network are robustly maintained even as the network is modified.

CME is a relatively well-defined pathway for clathrin-coated vesicle formation, but it connects to both upstream (receptor activation) and downstream (trafficking) pathways, and several proteins in this network participate in additional pathways. HSC70/HSPA8 plays an essential role in clathrin cage uncoating, but it also plays other roles in the cell as a chaperone, engaging in multiple additional interactions^8^. A valuable comparison would be to perform the stoichiometric analysis with copy numbers that are adjusted to account for how many proteins typically are free to participate in CME vs competing pathways. Without an objective means of performing this adjustment, we deferred here to the total abundances. For relative stoichiometries calculated here, such an adjustment would only have an impact if some proteins had orders-of-magnitude reductions in abundances, while others did not change. A reduction by all proteins of say 50% would not alter relative abundances.

We find several trends in the abundance and stoichiometry of distinct protein classes that are conserved across all 3 cell types. The proteins that assemble into rigid-like structures, actin (ACTB, ACTG1) and clathrin, are highly abundant, as are their respective disassembly proteins, cofilin (CFL1) and HSPA8/HSC70. Relative to the network of protein binding partners, these proteins are nearly always super-stoichiometric, or in excess supply. In contrast, enzymes have low abundance, and are nearly always sub-stoichiometric, or in demand. We speculate that this is representative of more general control mechanisms throughout the network, where proteins with low abundance act as control nodes or gatekeepers that determine when or where assembly occurs. There is essentially a reservoir of clathrin and actin available to assemble, and a reservoir as well of their corresponding disassembly factors. Although these abundant proteins self-assemble, the clathrin interactions in particular are weak (> 100 μM)^75^, and the clathrin-adaptor interactions are also typically weak^66^. With a reservoir of abundant proteins, the timing of these events is therefore controlled by essential co-factor proteins, as is the case for clathrin disassembly, which requires the DNAJC6/auxilin or GAK proteins that have low abundance and sub-stoichiometry. Experimentally, it was shown that this clathrin disassembly machinery is found to localize to complete vesicles, not the pre-fission coated pits^76^. Sub-stoichiometry cannot ensure that co-factors will never localize to sites of clathrin-coated pits, and indeed it is not known if other factors are important in preventing any disassembly by HSPA8/HSC70 prior to membrane budding, as these binding interactions to clathrin are not competing with other partners. However, by estimating the footprint of a single clathrin within a lattice as ~ 0.0009 μm^2^, even if 40% of the plasma membrane of a HeLa cell was coated in clathrin, more than half of all clathrin trimers would still be in solution. While HSPA8/HSC70 is sufficiently abundant to readily localize to clathrin in coated pits, the sub-stoichiometric co-factors would certainly help minimize disassembly at the plasma membrane.

Understanding the temporal sequence of arrivals and binding events in CME is not possible with the stoichiometric balance approach here, as we effectively solve for an equilibrium solution. As described in the introduction, a dynamic model with a complete set of binding affinities and rate constants for all interactions is necessary to describe such a sequential formation of distinct complexes, and is intractable for a process of this type and scale. The arrival of proteins like FCHO1/2 are expected to be early in clathrin-coat assembly^10^, forming interactions with the AP-2 appendage domains that are subsequently displaced as FCHO1/2 but not AP-2 becomes localized to the outer edges of clathrin-coated structures^77^. Explaining this type of behavior is not possible by our analysis of binding networks and copy numbers, and is therefore an important future work for a more detailed dynamical model. Our model ultimately quantifies total supply and demand for a protein given its interfaces and binding partners, independent of temporal events. As we previously noted, if local copy numbers of a protein were significantly different than average cellular abundances, the expected stoichiometry would therefore vary from our predictions. Similarly, if a protein interface with multiple binding partners had widely varying affinities for these partners, this would also shift the stoichiometry compared to our model predictions, biasing complex formation towards the most stable interaction. For many of the protein interfaces such as SH3 domains, we do not expect this to be a significant issue, as their binding partners are SLiMs that are structurally and chemically similar to one another, and affinities are not expected to vary widely outside of the low μM regime^65^. For some interfaces, however, they have a chemically and structurally diverse set of partners, such as actin and the PH-ear domain of NECAP1 as shown in Fig. 2B. In these cases, the affinities could be the more dominant factor determining complex formation rather than abundances, and therefore the stoichiometry of these specific binding interactions should more effectively be weighted to reflect this variability in binding strength.

Overall, we find that identifying imbalances in stoichiometry across a large protein binding network provides a systems-level perspective for contrasting how supply and demand for each individual protein either match up with or greatly deviate from their known interaction partners. The curated interface-resolved network provides a resource for future studies, as changes in specificity of individual binding interactions can be compared throughout evolution^24^, across diseases^30^, and changes in network connectivity can be compared against the interface-resolved network in yeast^24,51^, for example. Stoichiometry can also be assessed under a range of distinct perturbations than those tested here, including protein overexpression, which occurs in specific diseases, or removal of individual interfaces, to test a response to a more targeted perturbation via, e.g., small molecule inhibitors. While identifying imbalances as we do here does not explain why imbalances exist, they nevertheless can suggest promising avenues for follow up. For instance, it is perhaps not surprising that several proteins in the synaptosome have distinctive abundances, considering it is like a compartment at the end of a neuronal cell that contains mitochondria but not a nucleus^5^, and thus does not perform all of the same functions as a cell. SYNJ2 and endophilin (SH3GL2) have increased by 800 and 500 fold, respectively, but they play new key roles in ultrafast endocytosis^78^ in synaptosomes, which is not used in HeLa cells (although clathin-independent endocytosis can use endophilin even in non-neuronal cells^79^). Other dramatic changes could be similarly linked to alternate functional roles, and to our knowledge, it was not previously recognized how this dramatic increase in the lipid phosphatase SYNJ2 is also accompanied by a similarly dramatic increase in the lipid kinase PIP5K1C that reverses its action, which would help with membrane homeostasis. Given the revolutions in structure determination of large protein complexes and the proteomics scale resources on protein abundances across organisms^6,7^, the SBR analysis performed here offers a novel quantitative approach to integrate these molecular and cellular datasets.

## Methods

### Network construction

To construct the mammalian CME interface-resolved network, we initially considered 86 proteins. These 86 cytoplasmic proteins were selected due to their functional importance to the endocytosis pathway based on a systems-level study^10^, a comprehensive review ^11^, as well as homology to proteins in the yeast interactome^51^. To begin constructing our interface-resolved PPIN, we pooled unique PPIs mediated by only proteins listed in our original 86-protein CME list, downloaded from 3 open-source online molecular interaction repositories compiled from comprehensive curation efforts: Biological General Repository for Interaction Datasets^56^, IntAct^57^, and Mentha^58^. BioGrid provided the highest number of reported PPIs. IntAct is the only database that also curates information on domain resolution when possible.

Our completed interface-resolved human CME PPIN contains 82 proteins and 617 edges. Four of the original proteins either lacked reported PPIs in databases or lacked domain resolution for interactions. Of the 617 edges assigned, 433 were identified by a repository and interfaces were assigned with sufficient literature evidence, and 183 edges were added by manual curation, primarily due to studies on interactions with highly homologous proteins from other mammals (see Table S1 for % homology). We removed 31 edges due to suspected indirect and/or false interactions, and 202 edges downloaded from the repositories were left unassigned due to insufficient literature evidence to define binding sites. Overall, our filtered interface-resolved mammalian CME PPIN contains 82 proteins, 5 phospholipids, 9 transmembrane cargo groups that include 31 unique cargo proteins, and 396 distinct interfaces across all proteins and lipids. Our transmembrane cargo groups are YXXØ_class (where Y is tyrosine, Ø is a bulky hydrophobic residue and X is any residue), low density lipoprotein (LDL) receptors/phosphotyrosine binding domain targets (PTB_class), ubiquitylated cargo (ubiq_class), specific VAMP proteins (VAMP2 and VAMP7) and a broader set of VAMPs (SNARE_class), the receptor GPRC5A (labeled GPCR), and the receptor SYT1 (not present in HeLa or fibroblast) as their adaptor partners are known or inferred from sequence homology, with the complete list and classification in Table S1.

Below, we define the protocols and rules we used for delineating interface-resolved PPIs, given sufficient data, and rules for distinguishing interfaces within overlapping binding regions (Fig. S1). Domains that mediate a PPI could be assigned based on varying levels of evidence, listed in descending order of definitiveness: both identified via co-crystal structure; both identified with residue information derived from in vivo and/or in vitro experiments; one identified and the other has been inferred from mammalian or yeast homology; one identified with supporting in vivo and/or in vitro evidence, and the other speculated; both inferred; and both speculated. The most straightforward assignments are based on crystal structures of the human proteins in co-complex with one another, with the majority of assignments, however, being based on biochemical methods between either the human proteins, or mammalian and yeast homologs.

#### Assigning interfaces to PPIs

We document relevant details, e.g. PubMed IDs corresponding to studies sourced, and justifications for binding interactions in Table S3, as we individually annotate each PPI pulled from the repositories. We kept two separate lists of mammalian (including *Bos taurus, Gallus gallus, Mus musculus,* and *Rattus*) and yeast homologs for each protein, along with their percentage values of sequence identity relative to their human counterparts determined from BLAST^80^. We collected homologs that reached a sequence identity score ≥ 60%.

We constructed a decision tree which we iterated through for every PPI per protein in constructing our interactome (Fig. S1). In brief, we first chose a protein and pooled its unique PPIs across the 3 databases. Starting with one of its PPIs, our assignment task starts with a question that asks for evidence of highest specificity derived from a co-complex crystal structure, if available. Otherwise, the decision-making process continues with a series of questions that descends in orders of definitiveness, from residue level experimental support to: (1) whether domain information is available for both proteins or either protein; (2) whether domains for both or one of the proteins could be inferred from yeast or mammalian homology referring to our lists of BLAST scores; (3) whether domains for both or one of the proteins could be speculated; and (4) if neither domain could be speculated, whether literature support for PPIs were derived from high-throughput (HT) methodologies such as affinity capture-mass spectrometry (AC-MS)/MS and yeast-two hybrid (Y2H) assays. Hence, this process required substantial manual curation of the literature.

In addition to careful annotation of each PPI, we kept track of all the domains per protein. In order to define interfaces for an interaction, we either had to select one of the interfaces we had determined so far or add a new interface. We began with interface assignment with those determined from SMART^50^. In some cases, these domains mediated more than one PPI, hence containing multiple binding sites based on residue subsets (see “Splitting binding domains into multiple binding interfaces” section, below). Interfaces could thus be broadly classified as structured (or globular “Glob.”) domains, specific residues within a structured domain, or short stretches of residues that were primarily part of unstructured regions, which are labeled Short LInear Motifs (SLiMs)^81^. In the process, we actively updated the domains of the partner proteins.

#### Matched edges

Of the 617 edges in our PPIN, 443 are marked “MATCHED”. These interactions were identified by at least one of the 3 repositories and were retained in the network given literature evidence supporting a direct and specific interaction.

#### Splitting binding domains into multiple binding interfaces

Specific binding sites and interfaces often lie in large, spanning regions that define an entire folded domain. These domains were split if residues have been reported to mediate the interaction. We do this because partners for a spanning region of a protein can be noncompetitive, meaning both interactions can simultaneously be formed, and because not all residues are important for specificity of the interaction. We do not verify that all distinct interfaces are sterically accessible simultaneously, as done in one study^32^, focusing instead on the specificity of the individual interface residues for distinct partners, similar to previous work^51^. PRDs provide an example of unstructured regions containing multiple sequential binding motifs with often distinctive specificity for binding partners^82^. For example, the Huntingtin-interacting protein 1 (HIP1R) has a PRD including residues 1016 to 1068. The HIP1R interaction with cortactin (CTTN) specifically requires residue 1016, whereas mutations of essential residues in the 1025–1030 motif do not seem to impact binding. Residues 1025–1030 mediate specific binding of HIP1R to the three SH3 domains of SH3KBP1/CIN85, hence these regions are designated as distinct interfaces. BAR domain proteins provide an example of a structured domain with multiple binding interfaces. These domains can simultaneously form dimers with one another, bind to the membrane, and form higher-order oligomers. Thus, for each BAR domain, we created separate interfaces for dimerization, lipid binding, and oligomerization.

#### Specificity for distinct copies of repeated domains

Similar to the PRD example above, proteins also can contain repeated copies of a structured domain that have distinct specificity, requiring us to list each copy as its own interface. As an example, intersectin 1/2 (ITSN1/2) proteins each contains 5 unique SH3 domains, SH3A-E, that have distinct binding specificities to protein partners such as DNM2 and WAS.

#### Speculating interfaces for binding interactions

Many proteins in our CME list share the same protein binding partners and sometimes have shared binding regions. We use this information, coupled with homology, to define interface-resolved interactions into our PPIN, despite not having direct evidence of the specific domains used for the binding pair. To demonstrate our definition for speculating interactions, we use A1, A2, A3 isoforms of endophilins (SH3GL2/1/3 respectively), along with AMPH and BIN1, as they all share similar domain architecture and overlap in binding partners, to help define binding interactions to DNM1 and DNM2. We use homology to define SH3-PRD interactions that are known to DNM2-SH3GL1 for speculating binding interfaces for DNM1-BIN1 and DNM2-BIN1.

#### Added edges

In our curated PPIN, 184 new edges were added to account for interactions that were not present in the human PPI databases, but were supported by other literature studies. Of the 184, 80 of these added edges are lipid and cargo interactions, which we did not initially pull from the repositories. Most of the other added edges were not identified by any of the 3 repositories and were added during our re-evaluation of “MATCHED” PPIs via literature research. We sought to use homology for resolving interfaces of added PPIs, which we make note of below.

#### Using homology to add functional PPIs based on definitive biochemical evidence

Some interface-resolved “ADDED” PPIs were included using homology-based inference. These are interface-resolved PPIs informed by low-throughput biochemical experiments using homologous proteins. We take DBNL-DNM1 as an example. Mouse Actin-binding protein 1 (mAbp1), physically associates with rat Dnm1, serving to be a physical link between the actin cytoskeleton and endocytosis in both membrane transport processes in neuronal and non-neuronal cells at actin-rich sites, confirmed by immunoprecipitation and immunofluorescence microscopy within the same study. Additionally, because mouse Abp1 and human Dbnl share a sequence identity score of 85.39% and rat and human Dnm1 a high score of 98.03%, we thus added DBNL-DNM1 into our network.

#### Using homology to infer interfaces for dimerization and oligomerization

Since CME depends on dimerization and oligomerization of BAR-containing proteins such as F-BAR domain only proteins 1/2 (FCHO1/2), these PPIs were added into our network, if not already identified. For example, in our network, we identified FCHO2:FCHO2 as a “MATCHED” edge, but not FCHO1:FCHO1 nor FCHO1:FCHO2. However, the crystal structures of the yeast homolog SYP1 for human FCHO1/2 proteins and the structure of FCHO1 paralog FCHO2 homodimer have both been resolved. Using this information, we inferred interfaces for homodimerization and oligomerization interactions for FCHO1, adding the FCHO1:FCHO1 and FCHO1:FCHO2 PPIs into our network.

#### Inclusion of protein–lipid and protein–cargo interactions

Unlike other existing protein–protein interactomes, our interface-resolved network integrates relevant CME protein-lipid interactions as well as protein-cargo interactions (Table S3). Our inclusivity of lipid-binding interactions builds off of known identification of lipid-binding domains such as BAR, ENTH, and PH domains, including 42 of our proteins. Some of these interactions are enzymatic reactions that help regulate the population of phosphoinositides at the plasma membrane. For cargo interactions, although our interactome is not expected to be comprehensive in including all receptor cargos, we drew from reviews that classified types of cargo interactions specific to CME^60^.

#### Unassigned edges

202 edges were left unassigned from our network. High throughput Y2H- or AC-MS/MS-based screening used to construct large integrated protein interaction libraries PPIs can produce false positives and false negatives. If we could not find additional evidence to support an interaction between two proteins, this PPI was unassigned. Interactions that were unassigned were also those pulled from low-throughput experiments that lacked domain resolution.

#### Removed edges

31 edges were marked removed from our original list, as there was evidence from additional experiments that they were indirect or false. As reported in the construction and characterization study for the yeast CME interactome^51^ the PDB structure of the ARP2/3 complex^83^ was used to assign interfaces, and thus all other subunit contacts found in the 3 repositories were removed. 11 of the 31, or 35.5% of the removed edges, were Arp2/3 subunit interactions disregarded for this reason. PPIs were also removed if they were reported in a high-throughput study but proteins were biochemically shown to not interact. Phosphatidylinositol-4-phosphate 5-kinase type 1 gamma interacts with the β appendage of AP-2, not the α subunit (Table S3).

#### Copy numbers by cell type

Organisms contain many different cell types, each of which produces its own pattern of protein expression levels and in some cases distinct splicing isoforms of protein encoding genes. Our study collected copy numbers from rat synaptosome (compartment at the end of a neural synapse)^5^, mouse fibroblast^4^, and two human HeLa cell ^2,3^ studies. In all three cells, some of our proteins had no copy numbers reported. Proteins whose copy numbers were not reported in a study might be due to low natural abundance with expression levels undetectable. Therefore, for these proteins we used the Human Protein Atlas^6^ and Proteomics DB^7^, which report protein expression levels by tissue types, to help determine whether proteins with no reported copy numbers had unknown copy numbers, or zero copy numbers. Specifically, we looked for expression for the synaptosome proteins based on neuronal tissue (brain, cortex); for the fibroblast proteins based on bone marrow, stroma, soft tissue and skin fibroblasts; and for the HeLa proteins based on cervix, uterine, and squamous epithelial cells. Proteins with zero expression were excluded from subsequent stoichiometric balance analyses by removing the proteins from the network. If the databases indicated any expression level of a protein in a specific cell type, it was left in the network. Proteins that do not have constraints on copy numbers (“—“) thus can constrain the stoichiometric balance of their partners, but they are not penalized at all for deviating from their observed (unknown) copy number.

### Stoichiometric balance optimization

The Stoichiometric Balance Optimization of Protein Networks (SBOPN) algorithm has been described previously^39^. Briefly, the algorithm requires the network of interacting proteins, with interfaces resolved on their parent proteins. The solution of the number of complexes for each pair of binding partners can be formulated as the optimal of a quadratic function, with linear constraints. Specifically, the copy numbers of each interface are defined as the sum over all complexes it is part of. Two soft constraints are applied. First, for a protein with multiple interfaces, there is a penalty to making them highly distinct from one another. Second, a protein’s copies can be constrained to a target value, typically the observed copies of a protein. There is one parameter, α, which controls which of these two soft constraints is more tightly applied, where a low value of α forces all interfaces to be the same within a protein. We use here a value of α = 1, which allows fluctuations in values across interfaces, similar to known variances in measured copy numbers. We also tested lower values of α, down to 0.001, which forces the interfaces on a single protein to have quite closely matched values (Fig. S6). The abundances for a protein then are on average not as tightly constrained to match the experimental copy numbers. With lower values of α, the trends on which proteins have higher and lower stoichiometry than one another are quite similar, but the magnitude of SBR values are shifted towards super-stoichiometry (Fig. S6). The other primary change with using small values of α is that with minimal variation in balanced copies of each interface, this source of sensitivity to knockdown of partners is removed. We focus our analysis on results obtained from α = 1, because it forces the protein abundances to be closer to those that are experimentally observed, and it allows protein interfaces to form wider variations in complex copies, being less restricted by a single interface that may have only a single exclusive partner. In one sense, this means that interfaces within a protein that form fewer complexes have effectively weaker interactions, and interfaces that form more complexes have effectively stronger interactions. Given the optimal number of complexes that are solved for, we can then calculate the balanced number of interface and thus protein copy numbers. The code is available on https://github.com/mjohn218/StoichiometricBalance, along with input files for this network and knockdown simulations. The interactive network file generated using Cytoscape v3.7.2^84^ is available as part of supplemental material. Pearson correlation coefficients (R) between distributions were always applied to log10 values of concentrations and distances.

We note there is a distinction in whether a protein has zero observed copies, or whether the number of copies is unknown. Proteins with unknown copy numbers remain in the network and are subject to stoichiometric balancing, but without any constraint on their target copy numbers. Those proteins not observed in a given cell type (according to the Human Protein Atlas and the Proteomics DataBase) are removed from the network for that cell type, given that they are not expressed (Table S1).

## Supplementary Information

Supplementary Information.

Supplementary Tables.
