# Supplementary material for: Integrating protein copy numbers with interaction networks to quantify stoichiometry in clathrin-mediated endocytosis
Source: Sci Rep. 2022 Mar 30;12:5413. doi: 10.1038/s41598-022-09259-w (PMC8967901; doi:10.1038/s41598-022-09259-w)
Supplement: Supplementary file 1 — Supplementary Information. [file 41598_2022_9259_MOESM1_ESM.pdf]

Supplementary Information

For

Integrating protein copy numbers with interaction networks to quantify stoichiometry in clathrin-mediated endocytosis

Daisy Duan[1], Meretta Hanson[1], David O. Holland[2], Margaret E Johnson[1]*

[1]TC Jenkins Department of Biophysics, Johns Hopkins University, 3400 N Charles St, Baltimore, MD 21218.
[2]NIH, Bethesda, MD, 20892.

Supplemental Figures S1-S12

Supplemental Table Legends S1-S6

Supplemental Tables S4-S6

Supplemental Figures

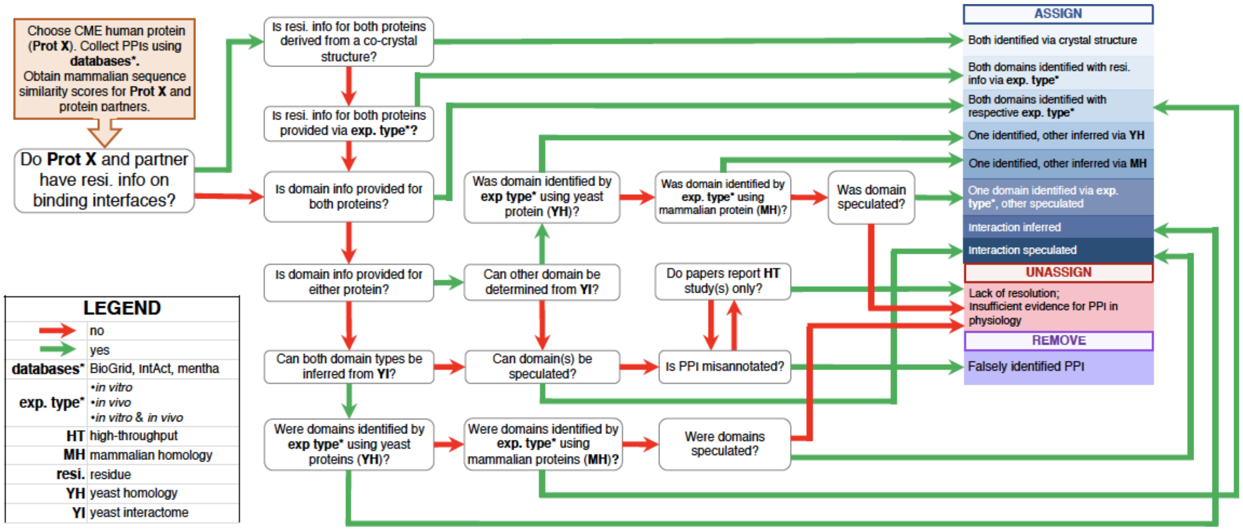

Figure S1. Decision-making workflow to annotate the assignment of a domain-resolved PPI based on experimental resolution. The decision-making series is followed by the network curator to determine whether the PPIs downloaded directly from BioGrid, IntAct, and mentha will be retained in the resulting CME network. There are interactions that differ in resolution based on the accuracy of the experimental methodology used to elucidate domain-resolved PPIs, in which case the decision-maker will delineate by listing specific experimental types used to elucidate them. With interactions that lack resi. and/or domain information, the user will be asked to infer domain partners of respective proteins using the YI curated by Johnson and Hummer (2013) with user's annotation detailing his/her inference of interacting domains based on YH. There are interface-resolved PPIs present in YI that have also been inferred using MH, in which case, depending on experimental type and whether domain information is provided for one or both proteins, the user will annotate with the appropriate level of experimental certainty, utilizing the provided BLAST sequence similarity scores of the human protein and its mouse/rat homologs. Interactions are speculated if no residue nor domain info is listed, but PPI is suspected to be mediated by two interfaces based on the domain architecture of the proteins and whether a similar interaction type is present in the CME network based on function. An interaction is unassigned if domain information is lacking. An interaction is removed if the PPI has been incorrectly annotated in the databases and is not physiologically functional.
exp. type: experimental type; **MH**: mammalian homology; **resi.**: residue; **YH**: yeast homology; **YI**: yeast interactome.

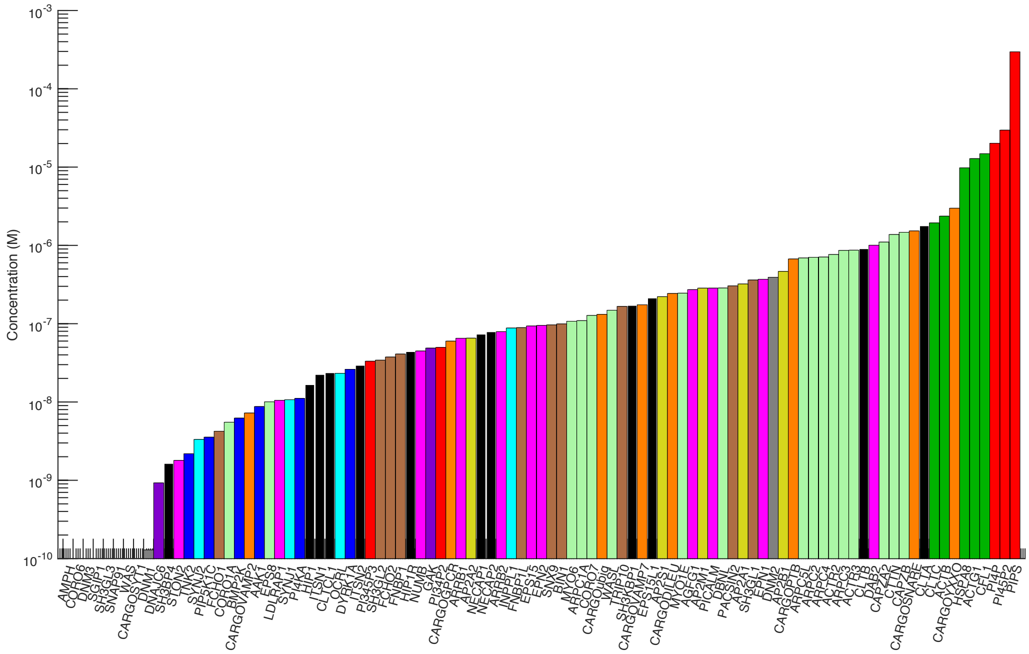

Figure S2. Concentrations of all HeLa proteins and lipids in the CME network range over 6 orders of magnitude. We include here lipids, which have cellular concentrations calculated by dividing copy numbers by the cell volume, with copy numbers defined based on lipid percentages on the plasma membrane[1]. DNM1 has the lowest concentration at 10^{-10} M, and the 8 proteins to the left of it are defined as having zero copies based on the Human Protein Atlas[2].

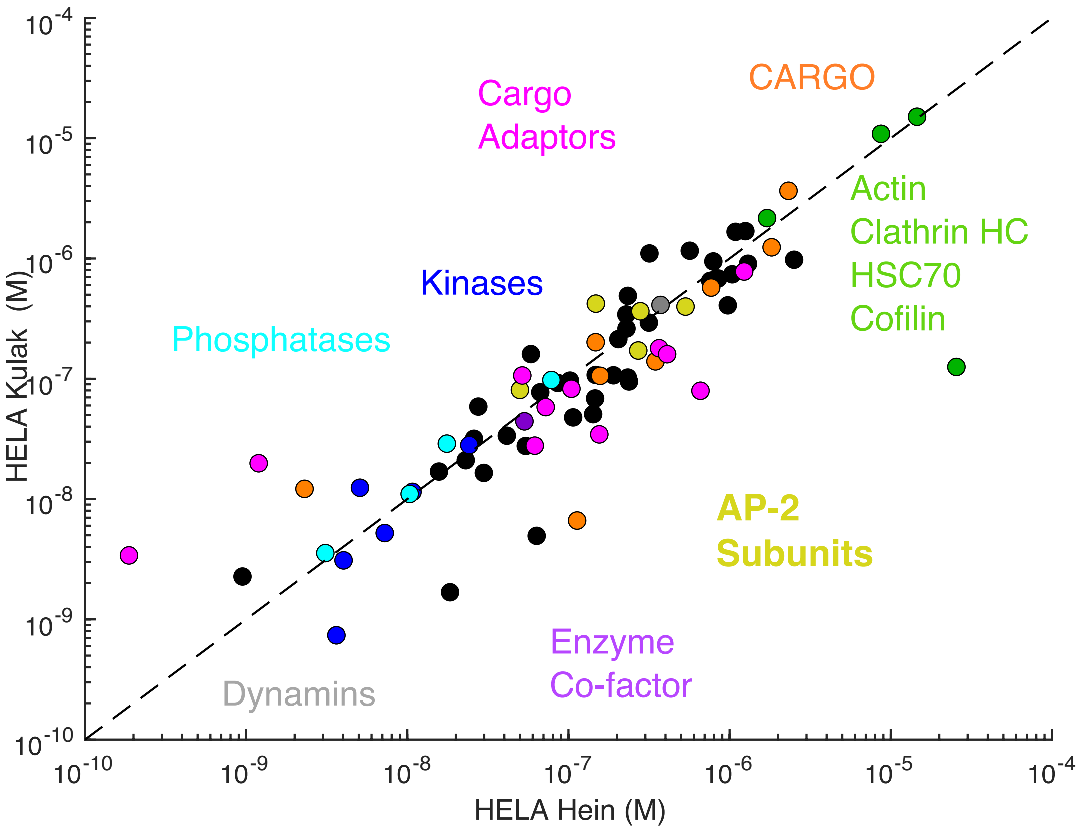

Fig S3: Correlation is high between CME protein copy numbers in two HeLa cell studies. Comparison between Hein et al[3], and Kulak et al[4]. We note that correlation is applied to the log10 values of the copy numbers, to identify correlations by order-of-magnitudes, giving R=0.83. Otherwise, on a linear scale, correlations are dominated by deviations between highly expressed proteins. The notable outlier is ACTG1, which differs by a factor of ~200 between studies. The dashed line indicates 1:1 correspondence (slope=1). For all HeLa calculations, we use the values averaged from these two studies. Proteins that were observed in only one study are not shown.

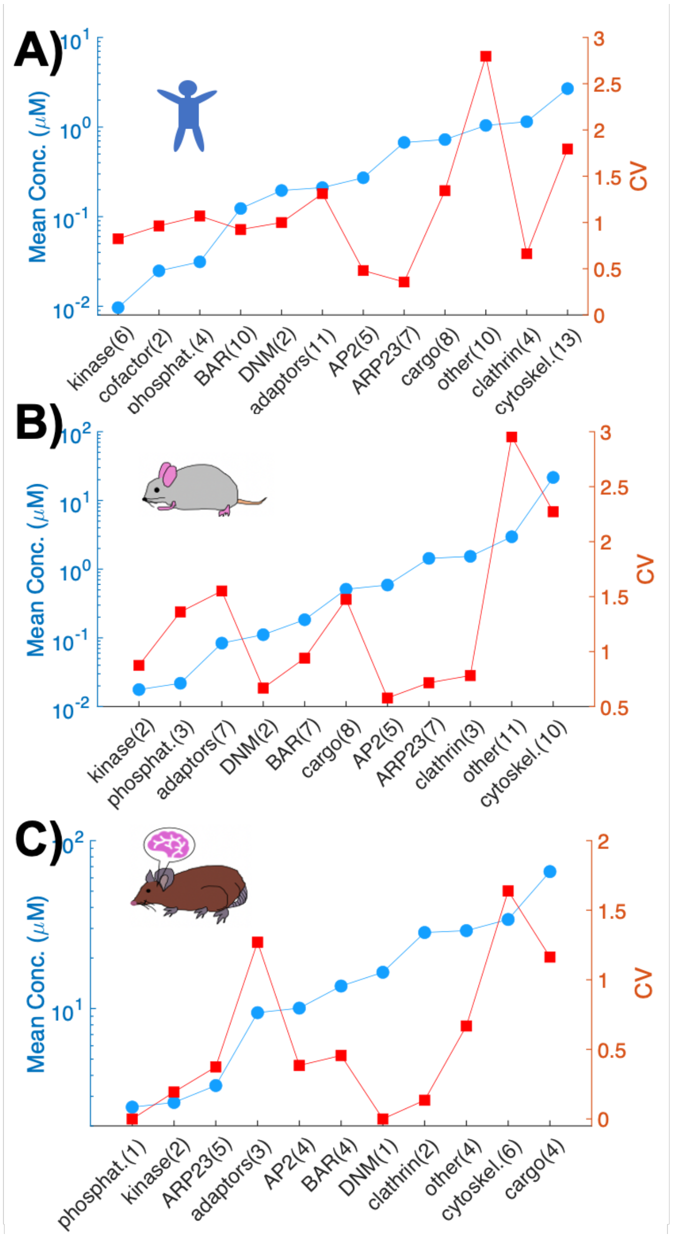

Figure S4. Protein copy numbers are least variable for subunits of multi-protein complexes. A) Proteins grouped by functional class are sorted according to their mean concentration (blue data) in HeLa cells. The number of proteins belonging to each group

are noted in parentheses. We quantified the coefficient of variation across the proteins in each group, $CV = \sigma/\mu$, where σ is the standard deviation and μ the mean of each group. The CV, (red squares) is lowest for the AP-2 and ARP2/3 complexes, and the clathrin trimer, which all have multiple subunits but function as an obligate complex. B). In fibroblast cells, the groups with the highest and lowest mean concentrations are very similar to HeLa, with enzymes on the low end, and cytoskeletal components on the high end. The CV is again low for AP-2, ARP2/3, and the clathrin trimer. C) The number of known abundances is significantly lower in synaptosomes, with the DNM (dynamin) and phosphatase groups having only a single known protein abundance. Overall, trends are similar to the other cell types. In all cell types, the lipids group (not shown) is 4-25 times higher than the most abundant group shown. Although the transmembrane cargo groups have higher mean concentrations than the adaptor proteins, they are biased by a few highly abundant groups, with each adaptor typically being more abundant than the cargo it selects for (Table S4-S6). The trends are weaker in the synaptosome, largely because the statistics were worse with far more unknowns: 49 proteins had unknown copy numbers versus 18 unknown in fibroblast and only 1 unknown abundance in HeLa. These persistent trends indicate that despite diversification of function amongst many distinct genes, the average supply of a protein does correlate with its function.

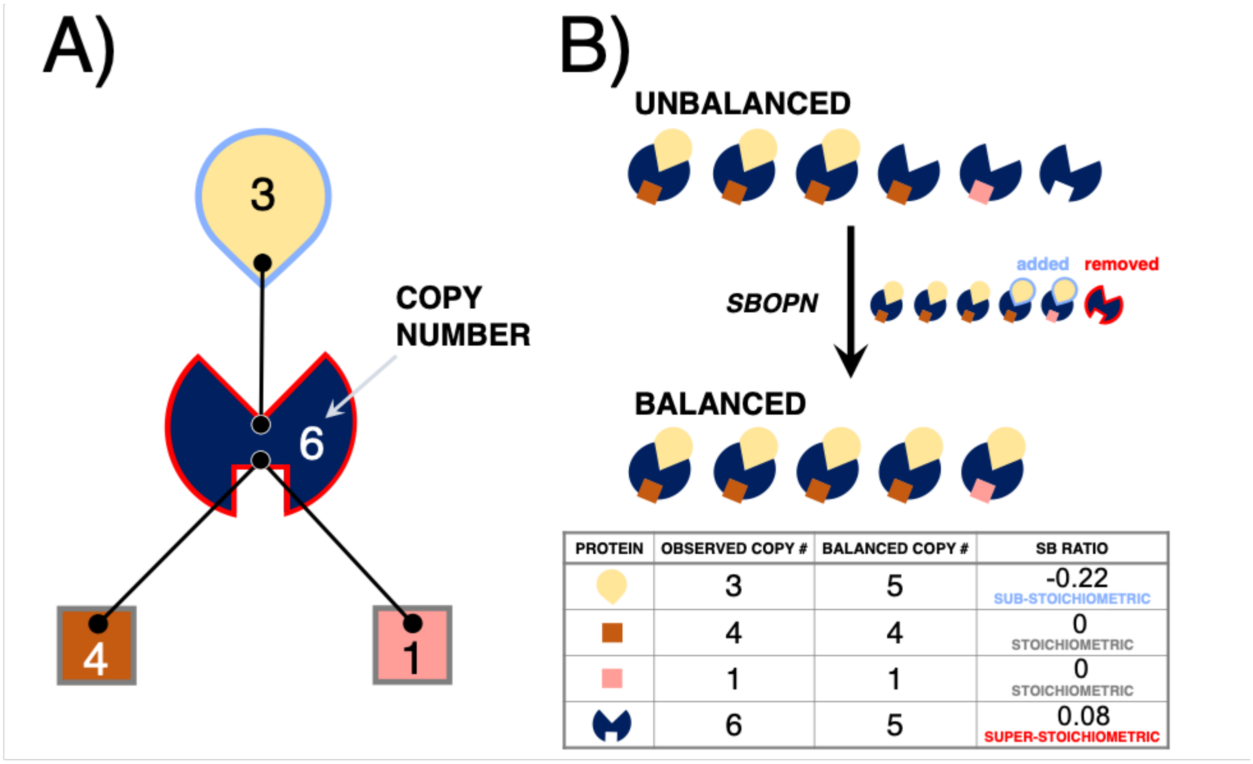

PROTEIN	OBSERVED COPY #	BALANCED COPY #	SB RATIO
(yellow)	3	5	-0.22 SUB-STOICHIOMETRIC
(orange)	4	4	0 STOICHIOMETRIC
(pink)	1	1	0 STOICHIOMETRIC
(navy)	6	5	0.08 SUPER-STOICHIOMETRIC

Figure S5. Illustration of the Stoichiometric Balance Optimization of Protein Networks (SBOPN) model applied to an interface-resolved network with observed copy numbers. (**A**) A simplified network diagram that contains four proteins, differentiated by color, where the central protein has two interfaces, and is thus capable of multi-component assembly. In the network, interfaces represented by black dots can only bind to their partners if their shapes are compatible, as reflected in nature where

protein binding involves conformational and chemical compatibility. Edges represented in black lines denote binding interactions. Observed copy numbers for each protein are shown in white/black text. (**B**) The observed copy numbers are unbalanced: several interfaces are unmatched. Note here that the square proteins compete for the same binding interface. Stoichiometric balance (SB) is achieved if all interface copies are matched to their partners and there are no leftovers. Here, the SBOPN algorithm removes an extra copy of the hub protein and adds two additional copies of the droplet-shaped protein to completely match all interfaces. To quantify SB, we calculate SBR = log10(observed copies/balanced copies), as illustrate in the last column of the table (Eq 1). We note that for multi-interface proteins (*i.e.* the blue one), not all interfaces must appear at the same copy number. When calculating SB ratio for a protein, we thus use the copies averaged across all interfaces.

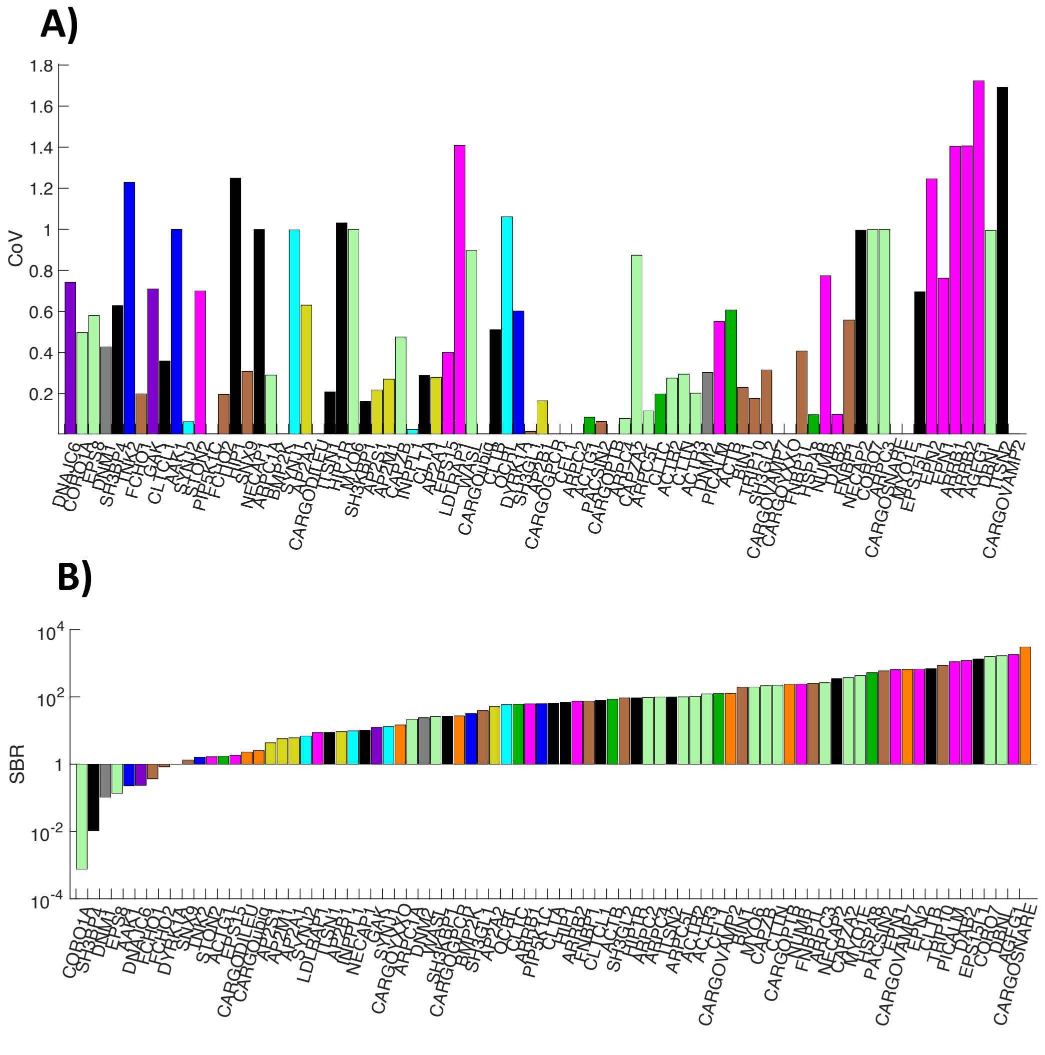

Figure S6. Variation in balanced copy numbers assigned across interfaces within a single protein are typically larger for adaptor proteins and enzymes. A) We calculated the coefficient of variation (CoV) for the balanced copy numbers assigned to each interface on a protein, CoV=σ/μ where the mean and the standard deviation are calculated for balanced copies assigned to each individual interface on a protein. Results

shown here for the HeLa cell copy numbers, corresponding to the SBR values in Fig 5E. For cargo proteins, the CoV is zero, because they each have only a single interface, and thus $\sigma=0$. B) We re-evaluated the SBR values of the HeLa cells (Fig 5E) putting a tighter constraint on having identical copies across each interface on a protein (we set $\alpha=0.001$, instead of 1 for Fig 5E). The trends from left to right are quite similar, but there is an overall shift up in stoichiometry, where now only 8 proteins are sub-stoichiometric. This result is due to having stricter limits on each protein: if one interface has to be kept to low abundance due to binding a partner with low copies, this affects all its other interfaces. This in turn affects all those interface partners, shifting most proteins to being super-stoichiometric. The CoV values for this data set (not shown) have similar trends to (A), but lower magnitude.

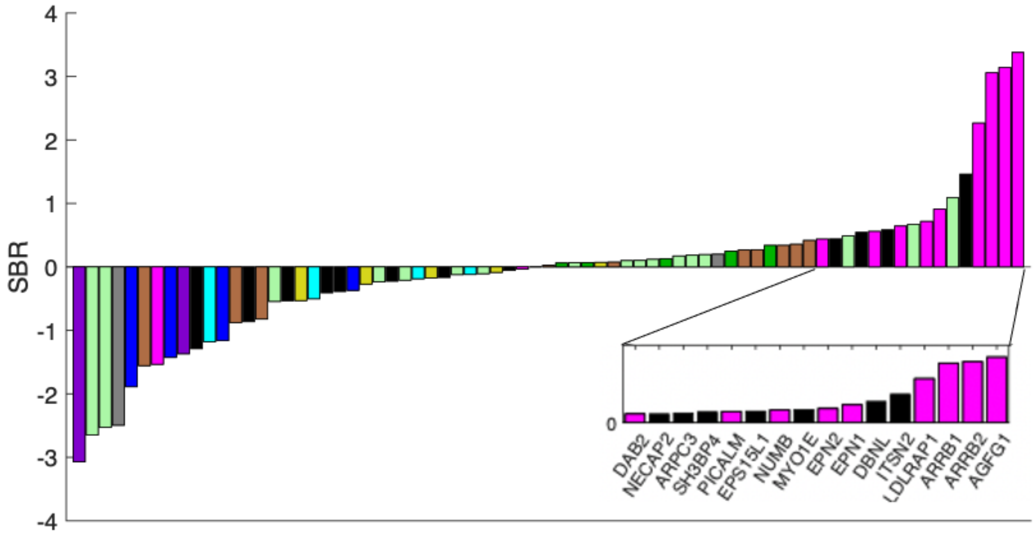

Figure S7. SBR values are similar when only cytosolic proteins are included without cargo. The major difference from Fig 5 of the main text (where both cytosolic proteins and cargo are included) is the scale of the SBR, with more dramatic super-stoichiometry observed for the adaptor proteins ARRB1, ARRB2, LDLRAP1, and AGFG1.

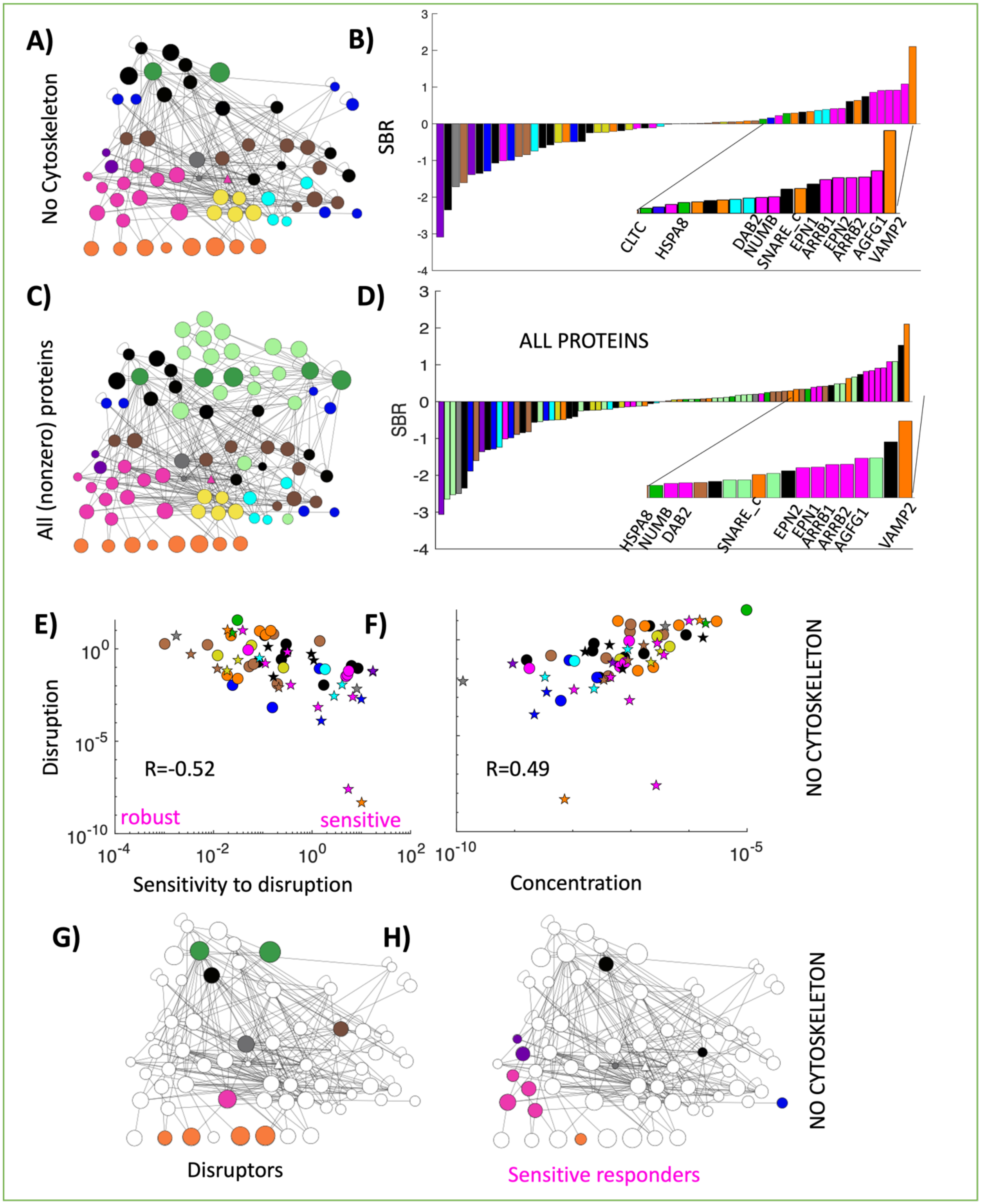

Figure S8. Stoichiometric analysis on the HeLa copy numbers with the cytoskeletal proteins removed shows similar results to the full network. A/C) HeLa protein network with nodes colored by type and sized by log10 abundance (see Fig 5 main text). All cytoskeletal proteins, previously colored in mint green, plus ACTB, ACTG1, and CFL1 (all dark green), have all been removed from (A). We also removed all edges connecting

to proteins with zero copy numbers (e.g. SNAP91, SH3GL3, DNM3, SYT1). B/D) The SBR values calculated without the cytoskeleton in (B) show very similar results to the full network SBR values in (D). For example, the adaptor proteins are still super-stoichiometric, along with several cargo, and the purple co-factors GAK and DNAJC6/auxilin are highly sub-stoichiometric. E-H) Results of perturbations performed on the network with no cytoskeletal proteins. E) Following removal of each protein from the network, their disruption (Eq. 3 main text) is anti-correlated with sensitivity (Eq. 4 main text), similar to the full network (see main text Fig 8). F) Disruption is also correlated with abundance. G) the most disruptive proteins are typically highly abundant, like HSPA8/HSC70 and DAB2, and several cargo. H) Sensitive proteins are typically more peripheral, such as PIP5K1C and the co-factors GAK and DNAJC6/auxilin. In this network, several of the more minimally connected adaptor proteins, such as AGFG1, ARRB1, ARRB2, and LDLRAP1 are also relatively sensitive to removal of their cargo, CLTC, or DAB2.

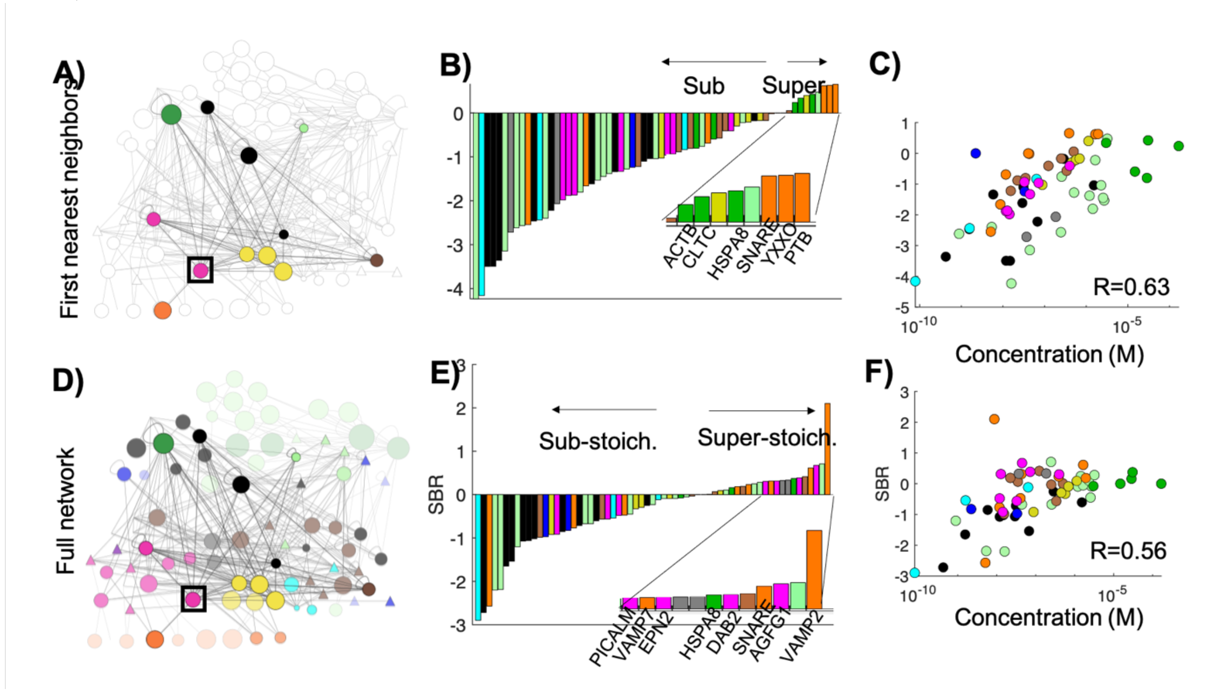

Figure S9. Stoichiometry in fibroblast of each protein to its direct binding partners, or to the full network, shows distinct patterns for highly abundant proteins. A) A simple model of balance compares a protein only to its immediate binding partners or nearest neighbors, shown here for cargo adaptor DAB2. Node size is scaled to log10(abundance). Triangles are proteins with unknown abundance. B) Competition for single interfaces results in most proteins being sub-stoichiometric, or without sufficient supply to meet partner demands. Stoichiometry is on a log10 scale, each integer thus indicating orders-of-magnitude differences between observed copies and balanced copies. C) Because only nearest neighbors are considered, partner stoichiometry correlates strongly with concentration, with abundant proteins most likely super-stoichiometric. D) With the stoichiometric balance method, not only first neighbors (dark nodes) but second (faded) and beyond (more faded) neighbors contribute to overall stoichiometry through their competition. E) With the SBR, many sub-stoichiometric proteins are similar to the simpler balance method. However, super-stoichiometric

proteins now include many of the cargo adaptor proteins (pink), which are all competing with one another to bind AP-2 and clathrin. F) The SBR has weaker correlation with concentration, as proteins with lower abundance are also super-stoichiometric. The cargo VAMP2 has low abundance but is super-stoichiometric, due to competing with the abundant SNARE class for internalization.

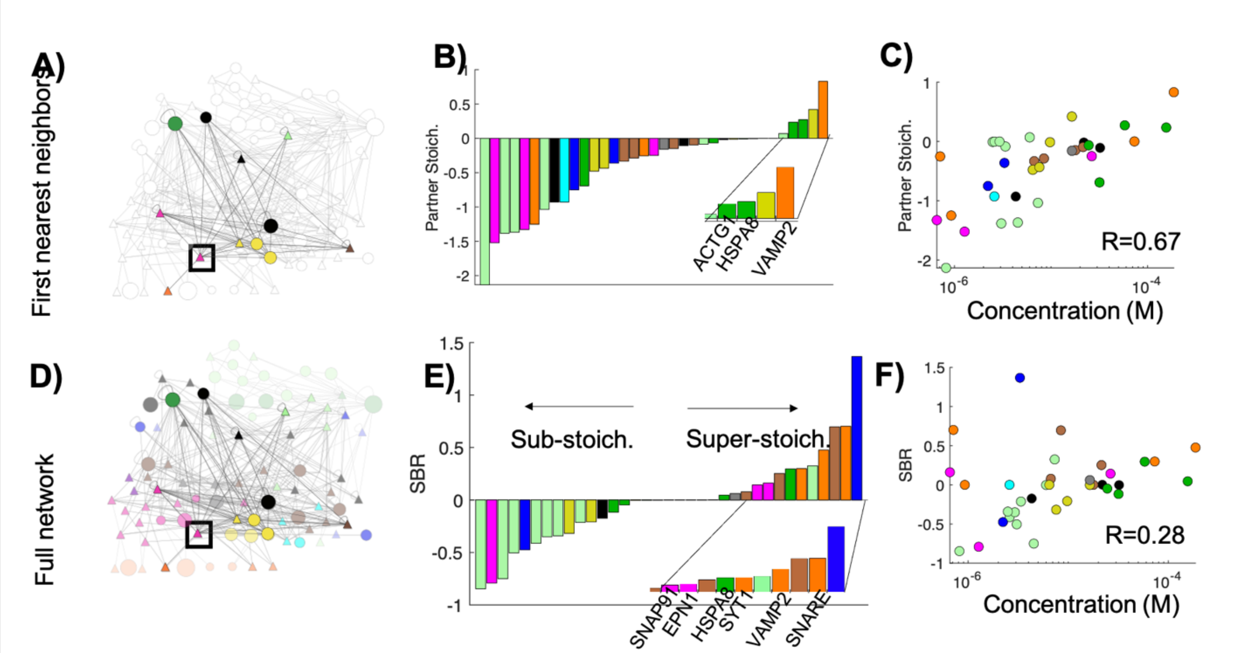

Figure S10. Stoichiometry in synaptosomes of each protein in to its direct binding partners, or to the full network, shows distinct patterns for highly abundant proteins. A) A simple model of balance compares a protein only to its immediate binding partners or nearest neighbors, shown here for cargo adaptor DAB2. Node size is scaled to log10(abundance). Triangles indicate proteins with unknown abundance, which are numerous in the synaptosome. B) Competition for single interfaces results in most proteins being sub-stoichiometric, or without sufficient supply to meet partner demands. Stoichiometry is on a log10 scale, each integer thus indicating orders-of-magnitude differences between observed copies and balanced copies. C) Because only nearest neighbors are considered, partner stoichiometry correlates strongly with concentration, with abundant proteins most likely super-stoichiometric. D) With the stoichiometric balance method, not only first neighbors (dark nodes) but second (faded) and beyond (more faded) neighbors contribute to overall stoichiometry through their competition. E) With the SBR, many sub-stoichiometric proteins are similar to the simpler balance method. Again, some of the cargo adaptor proteins (pink) are now super-stoichiometric in the SBR metric. F) The SBR has weaker correlation with concentration, as proteins with lower abundance are also super-stoichiometric, notably PIP5K1C. The cargo VAMP2 now has very high abundance, and is also super-stoichiometric. SNARE has lower abundance than VAMP2, but competes with VAMP2 to be internalized by PICALM, thus both cargo are in excess supply.

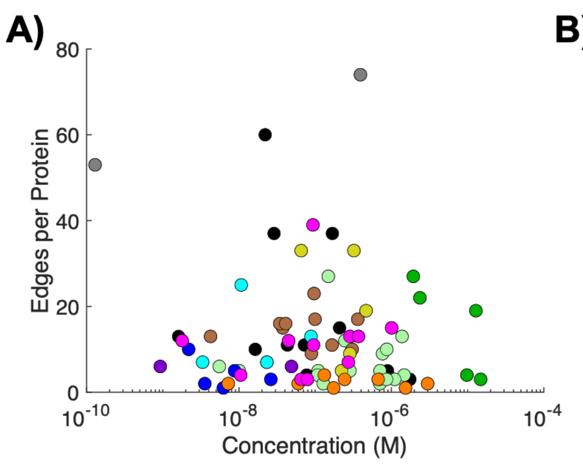

Figure S11. Edge numbers per protein have little to no correlation with abundance or stoichiometry. A) In HeLa cells, the correlation between edges per protein and abundance is R=-0.12. B) The correlation between edges per protein and SBR^2 tests whether highly connected nodes have an SBR closest to balanced or zero, and proteins with fewer connections can be sub or super-stoichiometric, which would produce a negative R value. The correlation is poor, with R=0.01. Similar trends occur in the fibroblast, with R=-0.09 and R=-0.02 for edge correlation with abundance and SBR^2, respectively. Correlation is higher in synaptosomes, with R=0.19 and R=-0.22.

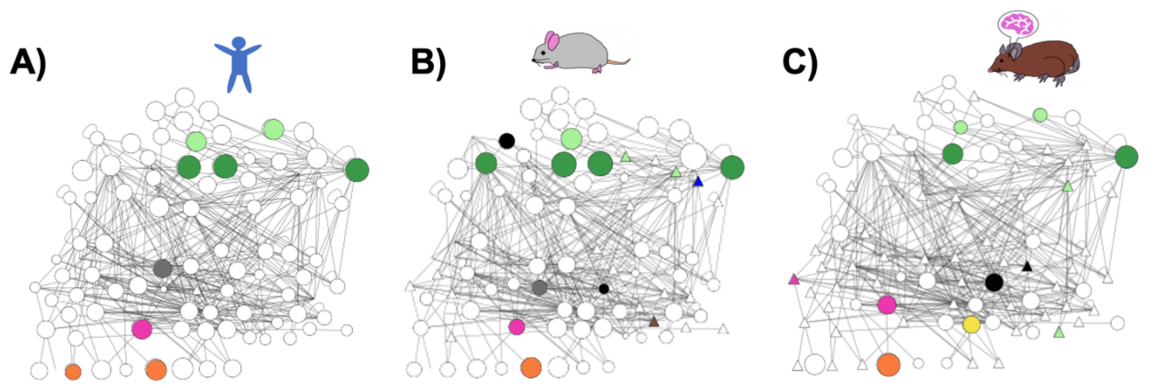

Figure S12. Proteins that are most disruptive upon removal are highly abundant and similar across cell types. A) Disruptors in HeLa B) Fibroblast C) Synaptosome. The highly abundant HSC70/HSPA8 is always disruptive, as are at least one cargo adaptor (Pink) and one cargo protein (orange). Some variations do exist, in part due to unknown copy numbers in fibroblast and synaptosomes (triangle shaped nodes). AP2M1 (yellow node) is more disruptive upon removal from synaptosome, as it is 20x higher than its cargo, whereas in the other two cell types, it is less abundant than its cargo. Node sizes are on a log10 scale, nodes with unknown copies are in triangles, and nodes with zero copies are points. Some of the biggest changes are specific adaptor/cargo from HeLa to

synaptosome. STON2 is a newly disruptive protein for synaptosome because of the receptor cargo SYT1, which is quite abundant but has zero copies in HeLa. Proteins with unknown abundance can sometimes be highly disruptive upon removal because they can absorb significant imbalances across the network without penalty.

TABLES:

Tables S1-S3 are in a separate excel file: SupplementalTablesS1-S3.xlsx

Table S1: List of all proteins, all cargo classes and the receptors assigned to each class, and all lipids, including copy numbers per cell type. Includes homology between human, rat, and mouse sequences. Includes annotation from the Human Protein Atlas and Proteomics DB about whether the protein is observed in specific tissue types.

Table S2: List of all domains and interfaces per protein that are within our interactome. Note that some proteins have additional domains, but we did not find that they mediated any interactions between our proteins. Including the known spans or approximate residues for each interface, when possible.

Table S3: List of all 617 interface-resolved interactions, including between proteins, proteins and cargo, and proteins and lipids. Also includes proteins interactions that we found in online databases, but could not assign interfaces to. Each interaction has interfaces (from Table S2) linked and listed, along with justification and publications cited.

TABLE S4: HeLa V=1455 μm^3 Bolded rows have cargo>adaptor

CARGO	*HeLa Conc (μM) (if cytoplasmic)*	*cargo Adaptor*	*HeLa Conc (μM)*
SYT1	0	STON2	0.0018
VAMP2	0.0072	PICALM+SNAP91	0.28+0
GPCR	0.0599	ARRB1+ARRB2	0.0652+0.0791
Ubiq	0.1315	EPN1,2,3+EPS15	0.37+0.095+UNK+0.0935
VAMP7	0.1744	AGFG1	0.2723
DILEU	0.2432	AP2A1+AP2A2+AP2S1	0.32+0.065+0.22
PTB	0.6708	DAB2+LDLRAP+NUMB	1.005+0.011+0.045
**SNARE**	**1.5311**	**PICALM**	**0.2848**
**YXXO**	**2.9863**	**AP2M1+SH3BP4**	**0.28+0.0016**

TABLE S5. Fibroblast V=1200 μm^3 Bolded rows have cargo>adaptor

CARGO	*Fibroblast Conc (μM) (if cytoplasmic)*	*cargo Adaptor*	*Fibroblast Conc (μM)*
SYT1	0	STON2	UNK
VAMP2	0.0086	PICALM+SNAP91	0.4+0
GPCR	0.04	ARRB1+ARRB2	UNK+0
Ubiq	0.012	EPN1,2,3+EPS15	0.012+0.013+0+0.033
VAMP7	0.04	AGFG1	0.04
DILEU	0.005	AP2A1+AP2A2+AP2S1	0.088+0.57+1.33
**PTB**	**0.39**	**DAB2+LDLRAP+NUMB**	**0.07+0.014+UNK**
**SNARE**	**1.66**	**PICALM**	**0.4**
**YXXO**	**1.93**	**AP2M1+SH3BP4**	**0.45+0.0004**

TABLE S6. Synaptosome V=0.235μm^3 Bolded rows have cargo>adaptor

CARGO	*Synaptosome Conc (μM) (if cytoplasmic)*	*cargo Adaptor*	*Synaptosome Conc (μM)*
**SYT1**	**73**	**STON2**	**UNK**
**VAMP2**	**187**	**PICALM+SNAP91**	**1.27+26.4**
GPCR	UNK	ARRB1+ARRB2	UNK+0
Ubiq	UNK	EPN1,2,3+EPS15	0.65+UNK+UNK+UNK
VAMP7	UNK	AGFG1	UNK
DILEU	UNK	AP2A1+AP2A2+AP2S1	UNK+6.46+9.76
PTB	UNK	DAB2+LDLRAP+NUMB	UNK+UNK+UNK
SNARE	0.7	PICALM	1.27
YXXO	0.92	AP2M1+SH3BP4	16.4+UNK

1 Yogurtcu, O. N. & Johnson, M. E. Cytosolic proteins can exploit membrane localization to trigger functional assembly. *PLoS Comput Biol* **14**, e1006031, doi:10.1371/journal.pcbi.1006031 (2018).

2 Cai, Y. *et al.* Experimental and computational framework for a dynamic protein atlas of human cell division. *Nature* **561**, 411-415, doi:10.1038/s41586-018-0518-z (2018).

3 Hein, M. Y. *et al.* A human interactome in three quantitative dimensions organized by stoichiometries and abundances. *Cell* **163**, 712-723, doi:10.1016/j.cell.2015.09.053 (2015).

4 Kulak, N. A., Pichler, G., Paron, I., Nagaraj, N. & Mann, M. Minimal, encapsulated proteomic-sample processing applied to copy-number estimation in eukaryotic cells. *Nat Methods* **11**, 319-324, doi:10.1038/nmeth.2834 (2014).
